# Self-Healing Polymer Nanocomposites: Mechanisms, Structure–Property Relationships, and Emerging Applications

**DOI:** 10.3390/polym18020276

**Published:** 2026-01-20

**Authors:** Sachin Kumar Sharma, Sandra Gajević, Lokesh Kumar Sharma, Yogesh Sharma, Mohit Sharma, Lozica Ivanović, Saša Milojević, Blaža Stojanović

**Affiliations:** 1 Surface Science and Tribology Lab, Department of Mechanical Engineering, Shiv Nadar Institution of Eminence, Gautam Buddha Nagar, Greater Noida 201314, India; 2 Faculty of Engineering, University of Kragujevac, Sestre Janjić 6, 34000 Kragujevac, Serbia; lozica@kg.ac.rs (L.I.); sasa.milojevic@kg.ac.rs (S.M.); blaza@kg.ac.rs (B.S.); 3 Department of Physics, GLA University, Mathura 281406, India; lokesh.sharma@gla.ac.in; 4 Department of Physics & Environmental Sciences, Sharda School of Engineering & Science, Sharda University, Greater Noida 201310, India; uvsbhu@gmail.com; 5 Department of Physics and Material Science, Jaypee University, Anoopshahr 203390, India; mohit.sharma@mail.jaypeeu.ac.in

**Keywords:** self-healing polymers, polymer nanocomposites, nanofillers, stimuli-responsive materials, structure–property relationships

## Abstract

Self-healing polymer nanocomposites are increasingly investigated as damage-tolerant materials for structural and functional applications; however, their engineering translation remains limited by the difficulty of achieving high mechanical reinforcement while retaining sufficient polymer mobility for effective repair. Previous reviews have largely summarized healing chemistries or nanofiller classes but have rarely established quantitative structure–property–healing relationships or resolved contradictory trends reported across studies. In this review, we develop an integrated framework that links polymer network architecture, nanofiller geometry/percolation behavior, and interfacial dynamics to healing kinetics, and we compile quantitative design windows for nanofiller loading, percolation thresholds, activation conditions, and durability metrics. The synthesis reveals that healing performance is maximized within intermediate filler contents near the percolation regime, whereas excessive nanofiller loading commonly suppresses healing by nanoscale confinement and interphase immobilization despite improving modulus and conductivity. Finally, we propose application-oriented design rules and benchmarking priorities, emphasizing standardized fracture/fatigue-based evaluation, multi-cycle healing retention, and scalable interphase engineering as the key pathways for translating self-healing nanocomposites from laboratory demonstrations to validated engineering systems.

## 1. Introduction

Polymeric materials are integral to modern technologies due to their tunable physical and chemical properties, low density, processability, and cost effectiveness. Consequently, they are widely employed in biomedical devices, flexible electronics, coatings, structural components, and energy-related systems. However, polymers are inherently prone to mechanical degradation mechanisms such as microcracking, abrasion, fatigue damage, and environmental aging, which progressively reduce performance and service life [1,2,3]. Even microscale defects can act as stress concentrators, accelerating failure under cyclic loading or aggressive operating conditions and thereby limiting the reliability of polymer-based systems [4,5]. Conventional damage-mitigation strategies, including material overdesign, external repair, and scheduled replacement, are increasingly viewed as inefficient and unsustainable, as they increase material consumption and maintenance costs while conflicting with current demands for durability and resource efficiency [6].

Self-healing polymers, inspired by biological systems capable of autonomous repair, have emerged as a promising approach to address these limitations by enabling materials to recover structural integrity and functionality after damage with minimal external intervention [7]. Since early demonstrations of autonomic healing based on encapsulated healing agents, research in this field has expanded to encompass diverse healing chemistries, polymer architectures, and activation mechanisms [8]. Self-healing polymers are generally classified into extrinsic systems, which rely on embedded healing agents released upon damage, and intrinsic systems, where healing arises from reversible interactions within the polymer network [9]. While extrinsic systems can provide effective one-time repair, their applicability is often constrained by limited healing capacity and long-term reliability concerns. In contrast, intrinsic systems enable repeated healing cycles and greater design flexibility, making them attractive for advanced polymer applications [10].

Despite notable progress, pristine self-healing polymers often exhibit persistent trade-offs between mechanical strength, healing efficiency, and functional performance. However, intrinsic self-healing is often limited by the well-known mobility–strength trade-off [11]. To address these competing requirements, increasing attention has been directed toward incorporating nanoscale fillers into self-healing polymer matrices, leading to the emergence of self-healing polymer nanocomposites. Nanofillers can improve stiffness, fracture resistance, thermal stability, barrier performance, and multifunctionality (electrical conductivity, photothermal conversion, and sensing) while also altering crack evolution and healing pathways through interfacial effects and energy dissipation [12].

During the past decade, several reviews have summarized intrinsic/extrinsic healing mechanisms and dynamic polymer chemistry, while others have discussed polymer nanocomposites in the context of reinforcement, transport properties, and functional integration [13,14,15]. However, a key nanocomposite-specific challenge remains insufficiently treated in a critical manner: nanofillers can simultaneously enhance healing (via crack bridging, improved heat/electron transport, and stress redistribution) yet suppress healing by reducing polymer chain mobility through nanoscale confinement, interphase immobilization, and network percolation [16]. This creates a fundamental contradiction, i.e., the percolation–mobility conflict where reinforcement and multifunctionality may be achieved at the expense of healability, especially under repeated damage–healing cycles and fatigue loading.

Accordingly, the novelty of the present review lies in providing an explicitly critical and analytical synthesis of self-healing polymer nanocomposites through a unified structure–property–healing framework. This work connects (i) nanofiller geometry, dispersion, and surface chemistry; (ii) interphase dynamics and confinement; (iii) electrical/thermal percolation and stimulus transduction; and (iv) healing kinetics, efficiency, and durability. In contrast to prior descriptive reviews, the present work consolidates quantitative design ranges and boundaries (typical filler loading windows, percolation thresholds, healing efficiency ranges, and repeatability metrics), clarifies mechanistic reasons behind conflicting literature outcomes (dispersion quality, interfacial chemistry, Tg proximity, and testing protocols), and proposes engineering translation metrics including fatigue-informed recovery, multi-cycle retention, and environmental durability. To clarify this positioning, Table 1 summarizes recent representative reviews and highlights the unique contribution of this work in enabling rational design of nanocomposites that balance reinforcement, multifunctionality, and reliable self-healing.

## 2. Fundamental Mechanisms of Self-Healing in Polymers

The capacity of a polymer to autonomously repair damage is governed by molecular-scale processes that enable crack closure, interfacial reconnection, and partial or complete recovery of mechanical and/or functional properties. These processes are strongly influenced by polymer chemistry, network architecture, chain mobility, and the nature of reversible interactions available within the material [25]. From a mechanistic perspective, self-healing polymers are commonly categorized as extrinsic or intrinsic systems depending on whether healing originates from externally stored agents or from reversible interactions inherently embedded within the polymer network [26]. This classification offers an important framework to understand healing behaviour, identify inherent limitations, and evaluate compatibility with nanocomposite design strategies.

Extrinsic self-healing polymers rely on healing agents that remain physically isolated from the polymer matrix until damage occurs. In such systems, crack propagation triggers the rupture of microcapsules or vascular networks, releasing a healing substance that infiltrates the damaged region and undergoes polymerization or curing to restore continuity [27]. Microcapsule-based approaches, where liquid monomers or reactive agents are dispersed throughout the matrix, have been widely investigated because they enable autonomic healing without external intervention [25,26,27]. Upon rupture, the released healing agent reacts—often with a catalyst incorporated within the matrix to form a repair phase that bridges opposing crack surfaces. These systems have been successfully demonstrated in thermosetting matrices such as epoxies and polyurethanes, where the concept of intervention-free repair is particularly attractive. However, despite their initial effectiveness, extrinsic systems suffer from intrinsic limitations: healing is often limited to one event per location due to finite agent availability, and capsule/vascular incorporation can introduce stress concentrations, reduce mechanical integrity, and complicate processing routes [2]. Consequently, extrinsic self-healing is generally more suitable for coatings, low-cycle damage environments, and short-life repair scenarios rather than for fatigue-dominated structural applications requiring long-term repeatability.

In contrast, intrinsic self-healing polymers derive their healing capability from reversible interactions within the network itself rather than consumable healing agents. Damage recovery occurs through reversible bond reformation, molecular rearrangement, or physical association of polymer chains across damaged interfaces [28]. Because the healing function is embedded into the polymer structure, intrinsic systems can provide multi-cycle healing, improved durability, and greater design flexibility, which is why they represent the primary focus of contemporary self-healing polymer research. A major intrinsic route is based on reversible covalent bonding, where dynamic covalent bonds reversibly break and reform under external triggers such as heat, light, or changes in chemical environment. Representative chemistries include Diels–Alder reactions, disulfide exchange, imine formation, and boronic ester dynamics [29]. These networks offer stronger interactions than purely physical systems, allowing for improved load-bearing capacity while maintaining healing capability. However, bond exchange often requires elevated activation conditions, limiting effectiveness under ambient service environments. Another important intrinsic class relies on supramolecular interactions such as hydrogen bonding, metal–ligand coordination, π–π stacking, and host–guest interactions [30]. These interactions are typically weaker than covalent bonds but exhibit rapid reversibility and frequently enable healing at room temperature or under mild conditions. Supramolecular systems therefore show fast healing kinetics for microdamage, although their mechanical robustness may be lower than that of covalently crosslinked networks [31]. Hybrid strategies combining multiple interaction types are increasingly adopted to achieve simultaneous strength and healability.

A further intrinsic pathway is observed in thermoplastic self-healing polymers, where healing proceeds through chain interdiffusion and entanglement across damaged interfaces when sufficient segmental mobility is activated [32]. Heating above the glass transition or melting temperature allows polymer chains to diffuse across crack planes, enabling gradual restoration of mechanical properties [30,31,32]. While conceptually straightforward, diffusion-driven healing typically requires significant thermal input and is frequently limited by slow kinetics and incomplete recovery under realistic service conditions. The strength–healing trade-off and its nanocomposite-specific origins are critically analyzed in Section 4.2. Importantly, in polymer nanocomposites, the dominant intrinsic healing response becomes strongly coupled with the type, geometry, dispersion quality, and surface chemistry of nanofillers, since these factors regulate interfacial interactions, local mobility, stress transfer efficiency, and activation pathways at damaged regions [33].

### Analytical Comparison of Extrinsic and Intrinsic Self-Healing Strategies

While extrinsic and intrinsic mechanisms are often presented descriptively, a practical engineering selection requires an analytical comparison based on repeatability, activation requirements, healing kinetics, scalability, and failure modes. Extrinsic systems typically exhibit fast initial healing due to agent release and polymerization, but their healing capacity is finite and decreases as capsules rupture or reservoirs deplete [32]. Their dominant failure modes are therefore associated with healing agent depletion, incomplete crack filling, reduced bonding quality of the healed interface, and mechanical weakening due to capsule-induced defects. In contrast, intrinsic systems enable repeatable healing cycles because network reversibility persists throughout the material; however, their limitation is typically healing kinetics, which depend on segmental mobility, activation barriers, and service-compatible triggering conditions [28]. Thus, for long-life structural performance and fatigue tolerance, intrinsic systems are often more suitable, whereas extrinsic systems remain attractive where rapid one-time repair is sufficient and manufacturing constraints permit agent encapsulation [34]. From a nanocomposite perspective, intrinsic systems offer additional advantages because reversible chemistry can be engineered at polymer–nanofiller interfaces, enabling nanofillers to contribute actively to healing (via crack bridging, localized energy dissipation, or trigger delivery) [16]. Conversely, high filler confinement may hinder diffusion-based healing if mobility is excessively restricted [35]. Therefore, intrinsic/extrinsic selection in nanocomposites must be guided by failure-mode compatibility under repeated cyclic loading rather than by healing efficiency reported from a single cut-and-heal event. A comparative analytical overview of both strategies and their dominant constraints is provided in Table 2.

## 3. Role of Nanofillers in Enhancing Self-Healing Polymer Systems

Nanofillers influence the strength–healing balance through three dominant pathways: (i) reinforcement and load transfer, (ii) percolation-enabled stimulus transduction, and (iii) interphase confinement, which together define the design boundary discussed in Section 4.2. In pristine self-healing polymers, increasing chain mobility to facilitate repair typically reduces stiffness and strength [46]. Nanofillers mitigate this limitation by creating efficient load-transfer pathways via interfacial stress transfer, while the surrounding polymer network retains sufficient segmental mobility for bond exchange or chain diffusion. This partial decoupling of mechanical performance from healing capability is therefore a key advantage of self-healing polymer nanocomposites [47]. However, because the filler–polymer interphase simultaneously controls reinforcement and local mobility, performance gains depend on balancing mechanical constraint against dynamic healing activation rather than simply increasing filler loading.

**Figure 1:**
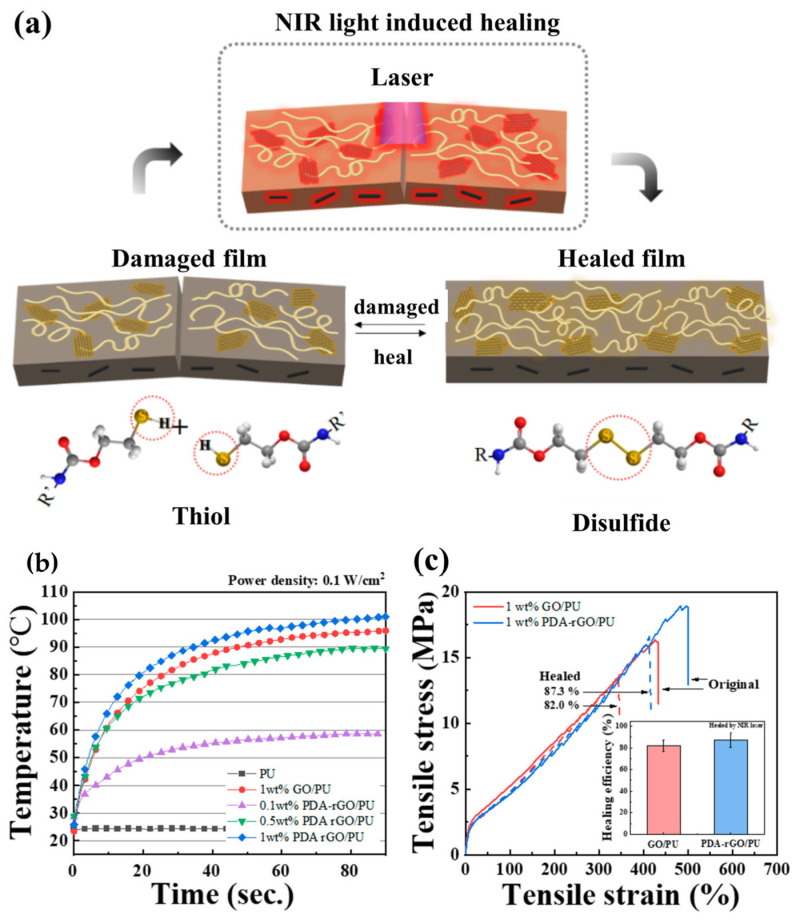
NIR photothermal self-healing in PDA-rGO/PU nanocomposites: (**a**) schematic of laser-induced healing via localized heating and thiol–disulfide exchange; (**b**) temperature rise under NIR irradiation (0.1 W cm^−2^) at different filler loadings; (**c**) tensile stress–strain response before/after healing with healing efficiency (inset) [49].

At the microscale, nanofillers strongly govern damage evolution by modifying crack initiation and propagation. Rigid or high-aspect-ratio fillers deflect advancing cracks, promote crack branching, and increase fracture energy, thereby reducing crack opening displacement and maintaining closer contact between damaged interfaces. This proximity is essential for intrinsic healing mechanisms driven by reversible bonding or diffusion-controlled re-entanglement, since it reduces the distance required for interfacial reconnection [48]. Figure 1 provides a representative evidence-based example of nanofiller-assisted healing [49]. Figure 1a illustrates NIR laser-induced localized repair in a PDA-rGO/PU nanocomposite where crack closure is activated by photothermal heating coupled with thiol–disulfide exchange. Figure 1b quantitatively confirms this activation pathway by showing a rapid and filler-dependent temperature rise under NIR irradiation, demonstrating how conductive nanofillers serve as localized thermal generators rather than passive reinforcements [49]. Figure 1c further links the mechanism to mechanical outcome, showing recovery in tensile stress–strain response after healing, with high healing efficiency values, thus supporting the argument that nanofillers can accelerate healing while retaining high stretchability and mechanical integrity. Together, these results highlight that nanofillers can act as healing activators by converting external stimulus energy into local thermal activation at the crack region.

In addition to crack geometry control, interfacial filler–polymer interactions strongly govern healing efficiency [50]. Functionalized nanofillers can participate in reversible covalent bonding, hydrogen bonding, ionic coordination, or supramolecular interactions with the polymer matrix, forming dynamic interphases that can break and reform during repeated damage–repair cycles. These dynamic interphases increase the density of reversible interactions and improve restoration of mechanical properties; however, excessively strong interfacial anchoring may suppress diffusion-dominated healing by immobilizing chains near the filler surface [51]. Accordingly, rational design of nanofiller surface chemistry is a critical parameter in the development of effective self-healing polymer nanocomposites.

Carbon-based nanofillers, particularly graphene derivatives and carbon nanotubes, are widely used owing to their exceptional modulus, high aspect ratio, and multifunctionality. Their strong load-transfer capability enables reinforcement at relatively low filler fractions, thereby limiting disruption of polymer mobility [52]. Furthermore, π–π interactions and surface functionalities can promote supramolecular interactions that support intrinsic healing pathways. Beyond reinforcement, conductive nanofillers impart electrical and thermal conductivity, enabling externally triggered healing through Joule heating or photothermal mechanisms [53]. Enhanced thermal transport is particularly beneficial, since many intrinsic mechanisms (dynamic covalent exchange, vitrimer rearrangement, and thermoplastic diffusion) require localized heating to activate repair kinetics [54]. Nanofillers with high thermal conductivity accelerate heat distribution at the damaged site, reducing healing time and improving recovery efficiency. This shift from passive reinforcement to active stimulus transduction is a defining contribution of conductive nanofillers in next-generation smart self-healing materials [55].

Inorganic nanofillers such as nano-clays, silica nanoparticles, and metal oxides provide additional reinforcement and functional routes. Layered silicates improve stiffness and barrier performance while offering high interfacial surface area for polymer–filler interactions [56]. When appropriately functionalized, these fillers can engage in hydrogen bonding or ionic interactions without severely restricting polymer mobility, supporting intrinsic healing performance. Metal oxide nanoparticles further expand functional scope through UV shielding, catalytic activity, or stimulus sensitivity (e.g., magnetic or photothermal activation) [57]. Notably, some metal oxides may also catalyze oxidative degradation depending on chemistry, dispersion state, and service conditions, suggesting that long-term stability considerations should accompany filler selection for engineering deployment [58].

Hybrid and multifunctional nanofillers represent an emerging strategy to simultaneously enhance mechanical performance, healing efficiency, and stimulus responsiveness. Combining conductive and reinforcing components can enable triggerable healing while preserving structural integrity, which is relevant for smart coatings, sensors, and flexible electronics [59]. Despite these advantages, nanofiller incorporation introduces challenges in dispersion, agglomeration, and processing. Poor dispersion can produce stress concentrators and reduce healing efficiency, whereas excessive filler loading may confine polymer chains and counteract intrinsic healing mechanisms [60]. Therefore, optimization of filler content and dispersion strategy is essential. From a design standpoint, the effectiveness of nanofillers depends not only on filler type but also on their spatial distribution near crack-prone regions and compatibility with the dominant healing mechanism [57,58,59,60]. Advanced processing routes, including in situ polymerization, solution blending, and surface grafting are increasingly used to achieve controlled nanostructures tailored for improved healing behavior [59,60]. A comparative summary of nanofiller influence on reinforcement, healing response, and multifunctionality is provided in Table 3. Overall, nanofiller effectiveness is maximized when filler selection, surface functionalization, and dispersion methods are co-designed in alignment with the underlying healing chemistry and intended activation strategy.

## 4. Structure–Property–Healing Relationships in Self-Healing Polymer Nanocomposites

Establishing robust structure–property–healing relationships is essential for the rational engineering of self-healing polymer nanocomposites that must simultaneously deliver mechanical reliability and effective damage recovery. In these systems, healing performance cannot be interpreted in isolation because polymer chemistry, network architecture, nanofiller geometry, dispersion state, and interfacial interactions collectively govern damage evolution and post-healing recovery [66]. Accordingly, optimizing a single parameter (e.g., filler content or crosslink density) rarely yields universal improvement; instead, performance is governed by coupled and often competing mechanisms. At the molecular scale, polymer network architecture provides the baseline healing capacity by controlling segmental mobility and bond exchange kinetics [67]. The interplay among crosslink density, interphase mobility, and healing kinetics therefore defines the achievable recovery envelope, as critically synthesized in Section 4.2.

Nanofiller geometry (size and aspect ratio) strongly affects stress transfer, crack behavior, and the development of conductive/thermal networks. High-aspect-ratio fillers, including graphene and carbon nanotubes, can form percolated networks at relatively low loadings, enabling effective reinforcement while maintaining polymer continuity [68]. These networks redistribute stress near crack tips, reduce crack opening displacement, and promote crack deflection—conditions that favor intrinsic healing mechanisms requiring intimate interfacial contact. In contrast, spherical nanoparticles generally require higher loading fractions to achieve comparable modulus enhancement, which increases the probability of agglomeration and interphase-driven mobility restriction [69]. Therefore, filler geometry influences not only stiffness but also healing kinetics by determining whether the composite shifts toward reinforcement-dominant or mobility-dominant behavior. Dispersion quality represents another decisive structural factor controlling both mechanical robustness and healing reproducibility. Uniform dispersion maximizes interfacial areas, distributes stress more homogeneously, and supports consistent energy dissipation across the damaged region, thereby improving the probability of restoring interfacial interactions during healing [70]. Conversely, agglomeration introduces local stress concentrators that accelerate crack initiation and cause nonuniform strain localization, which can both reduce apparent healing efficiency and increase scatter between samples. Thus, dispersion is not merely a processing concern but a controlling variable in structure–property–healing correlations [71].

**Figure 2:**
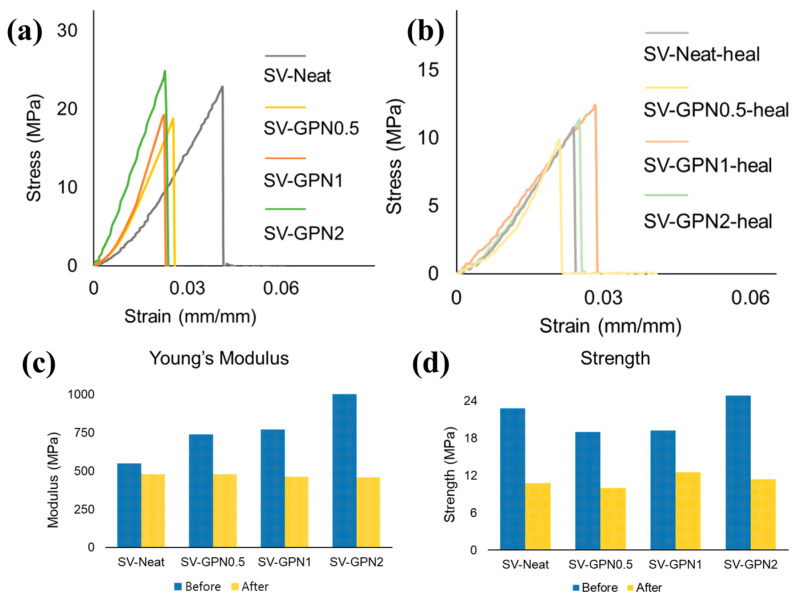
Mechanical performance of self-healing epoxy vitrimer composites with different GO-PANI (GPN) loadings: (**a**) stress–strain curves of pristine samples; (**b**) stress–strain curves after healing; (**c**) Young’s modulus before and after healing; and (**d**) tensile strength before and after healing, highlighting the reinforcement–healing trade-off with increasing nanofiller content [74].

Interfacial bonding between polymer chains and nanofillers ultimately dictates whether reinforcement and healing can be balanced or become antagonistic. Weak interfaces can result in filler pull-out and inefficient load transfer, whereas excessively strong or irreversible bonding can immobilize polymer chains in the interphase, suppress diffusion or bond exchange, and lower healing efficiency [72]. Dynamic or reversible interfacial interactions—including hydrogen bonding, metal–ligand coordination, and dynamic covalent exchange—are therefore particularly advantageous because they enable stress transfer during loading while still allowing for interfacial rearrangement during healing [73]. Figure 2 provides quantitative evidence of this interphase-controlled trade-off: Figure 2a shows that increasing GPN (Graphene Oxide (GO)–Polyaniline (PANI) loading enhances the pristine stress–strain response, confirming reinforcement. Figure 2b shows the healed stress–strain response, indicating that recovery becomes constrained at higher filler contents. Figure 2c demonstrates that Young’s modulus increases strongly with increasing filler fraction in the pristine state but does not recover proportionally after healing and Figure 2d shows a similar trend in tensile strength, highlighting incomplete restoration after healing due to interphase confinement and limited network rearrangement [74]. These trends support the conclusion that interphase engineering must preserve chain mobility and reversible bonding capacity rather than maximizing filler loading. Tailoring nanofiller surface chemistry (functionalization and compatibility) is therefore a key design strategy for tuning interphase dynamics and optimizing structure–property–healing performance.

Beyond chemistry and dispersion, spatial distribution of fillers also influences damage evolution and healing outcome. Fillers localized near crack-prone regions, interfaces, or stress concentrators can disproportionately alter crack growth pathways and promote repair by maintaining interfacial proximity [75]. In gradient or layered architectures, filler-rich regions can act as mechanically reinforced domains, while filler-lean regions retain higher mobility to enable fast healing. Such hierarchical spatial designs provide a rational route to reconcile strength–healing trade-offs by intentionally separating reinforcement and healing functions rather than forcing both into a single uniformly filled phase [76]. Thermal and electrical properties introduced by nanofillers further modulate healing behavior, particularly in stimuli-responsive systems. Higher thermal conductivity enables more uniform temperature distribution during thermally activated healing, reducing activation time and preventing thermal gradients that can generate residual stress [77]. Similarly, conductive filler networks enable localized Joule heating, allowing for selective activation at damaged regions without global heating of the component. These transport-driven structure–property linkages highlight the multifunctional role of nanofillers beyond mechanical reinforcement [78]. However, the emergence of transport networks often introduces a percolation–mobility conflict: as filler loading approaches percolation, activation efficiency increases but polymer mobility can be increasingly restricted [79]. This non-linear behavior is discussed analytically in Section 4.2 and implies that optimal filler concentration is system-specific and constrained within a narrow design window defined by polymer chemistry, filler type, dispersion quality, and the dominant healing mechanism. Finally, engineering relevance requires that structure–property–healing relationships be evaluated under repeated damage and long-term durability constraints. Structural features that provide high initial recovery may not sustain healing over multiple cycles if filler networks rearrange irreversibly, interfaces degrade, or network fatigue accumulates [80]. Consequently, recent studies increasingly emphasize assessing recovery under cyclic loading, fatigue crack growth conditions, and environmental exposure (humidity, temperature cycling, oxidation) to predict real-world reliability rather than relying on single-cycle tensile recovery [77,78,79,80]. Overall, healing performance in self-healing polymer nanocomposites is governed by an interdependent balance between polymer network dynamics and nanofiller-induced reinforcement. A correlation between key structural parameters and healing response is summarized in Table 4, emphasizing that successful design requires co-optimization of polymer chemistry, nanofiller geometry, dispersion state, dynamic interfacial interactions, and filler loading level. These considerations establish the foundation for stimuli-responsive self-healing nanocomposites, which are discussed in the following section.

### 4.1. Quantitative Design Ranges: Filler Loading, Aspect Ratio, and Percolation Thresholds

A central advantage of self-healing polymer nanocomposites is that healability can be coupled with multifunctionality and reinforcement by tuning quantitative nanofiller variables such as filler loading fraction (φ), aspect ratio (AR), and the onset of electrical/thermal percolation. Unlike conventional composites, where filler addition primarily aims to maximize stiffness or strength, self-healing nanocomposite design must operate within a constrained window where fillers provide crack-bridging and stimulus transduction without excessively suppressing polymer segmental mobility [84]. Therefore, extracting quantitative ranges from reported studies is essential to provide actionable design guidance and to rationalize conflicting results across literature.

At low filler concentrations, nanofillers mainly act as mechanical crack modifiers through crack deflection, crack pinning, and energy dissipation [16]. In this regime, stiffness and strength generally increase with minimal influence on intrinsic healing, particularly when filler–matrix interactions are weak-to-moderate and dispersion is uniform. However, as filler loading increases, a growing fraction of polymer chains becomes immobilized within the filler interphase, leading to restricted relaxation and reduced chain interdiffusion across crack planes [82]. This effect is most pronounced for high surface area nanofillers and strongly functionalized fillers, which can create a thick immobilized interphase. Consequently, healing efficiency often exhibits a non-monotonic dependence on filler loading: modest additions may enhance healing by improving crack closure and stress redistribution, whereas excessive loading reduces healing due to mobility suppression [85]. This is particularly important for intrinsic systems that rely on diffusion-driven healing or supramolecular rearrangement.

For conductive nanocomposites, percolation introduces a second, highly influential transition. Electrical percolation typically occurs at much lower loadings for high-aspect-ratio fillers (e.g., CNTs, graphene platelets, metallic nanowires) than for low-aspect-ratio fillers, enabling functional transport networks without high filler fractions [86]. Typical percolation thresholds for CNT-filled polymers are often in the range of ~0.1–1 wt.% depending on dispersion quality and AR, whereas graphene derivatives frequently require ~0.5–3 wt.% due to platelet restacking and aggregation tendencies. Once percolation occurs, conductivity can increase by several orders of magnitude, enabling Joule heating and rapid localized temperature rise under applied voltage [87]. This has direct implications for healing because localized heating accelerates dynamic bond exchange (e.g., vitrimer transesterification, Diels–Alder reversibility, disulfide exchange) and can restore mechanical performance in minutes rather than hours. Photothermal activation follows similar logic in systems containing graphene derivatives, MXenes, or plasmonic fillers, where light absorption enables localized heating [88]. Thus, percolation does not simply enhance multifunctionality; it provides an activation pathway that directly improves healing kinetics. However, high filler content beyond percolation can be detrimental. Above the percolation threshold, network densification increases confinement and reduces crack-plane mobility, lowering multi-cycle healing retention even if initial activation is fast [89]. Therefore, optimum designs typically target filler contents near the functional threshold: sufficiently high to enable transport-assisted activation but low enough to avoid network-induced immobilization. Aspect ratio plays a dual role as high AR reduces percolation thresholds but also intensifies confinement if dense entanglement networks form [90]. Accordingly, design should focus on achieving percolation via dispersion and AR optimization rather than by simply increasing filler loading.

Finally, the selected filler class must be matched to the intrinsic healing mechanism. Nonconductive fillers such as silica, alumina, nanoclay, and halloysite can improve modulus and crack resistance but do not directly enable stimulus transduction, and their influence on healing largely depends on interphase chemistry and mobility retention [91]. Conductive fillers provide powerful activation routes but require careful control of percolation and interphase design. Overall, quantitative ranges for filler loading, AR, and percolation threshold provide a practical framework to balance reinforcement, activation, and healing durability. Based on reported studies, Table 5 summarizes typical ranges and the dominant mechanical/healing outcomes across filler classes, serving as a design reference to guide future self-healing nanocomposite development.

**Figure 3:**
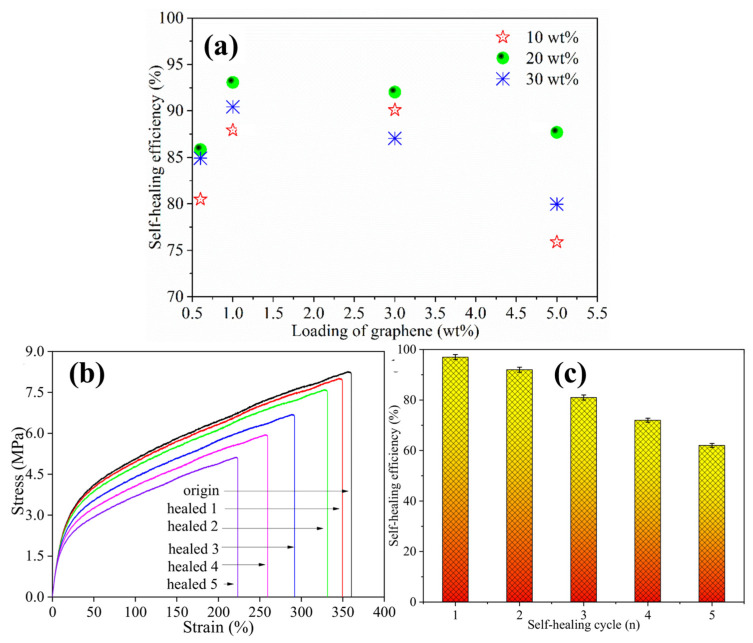
(**a**) Healing efficiency as a function of graphene loading for different TPU compositions (10, 20, and 30 wt%), (**b**) Stress–strain response of pristine and repeatedly healed samples (cycles 1–5), and (**c**) Healing efficiency versus healing cycle number (*n* = 1–5), showing performance degradation with repeated repair [97].

Quantitative evidence further confirms that healing efficiency in graphene/TPU nanocomposites follows an optimum loading window rather than a monotonic increase with filler content. As shown in Figure 3a, the healing efficiency increases up to ~1–3 wt% graphene (≈90–93%) due to enhanced photothermal heat generation but decreases at higher loading (≈76–88% at 5 wt%), consistent with nanofiller-induced chain mobility restriction and nanoscale confinement [97]. Multi-cycle recovery also degrades with repeated healing, as reflected by reduced mechanical extensibility in Figure 3b and the progressive decline in healing efficiency from ~97% (cycle 1) to ~62% (cycle 5) in Figure 3c. These results highlight the need to optimize filler loading for both initial healing and multi-cycle durability.

**Figure 4:**
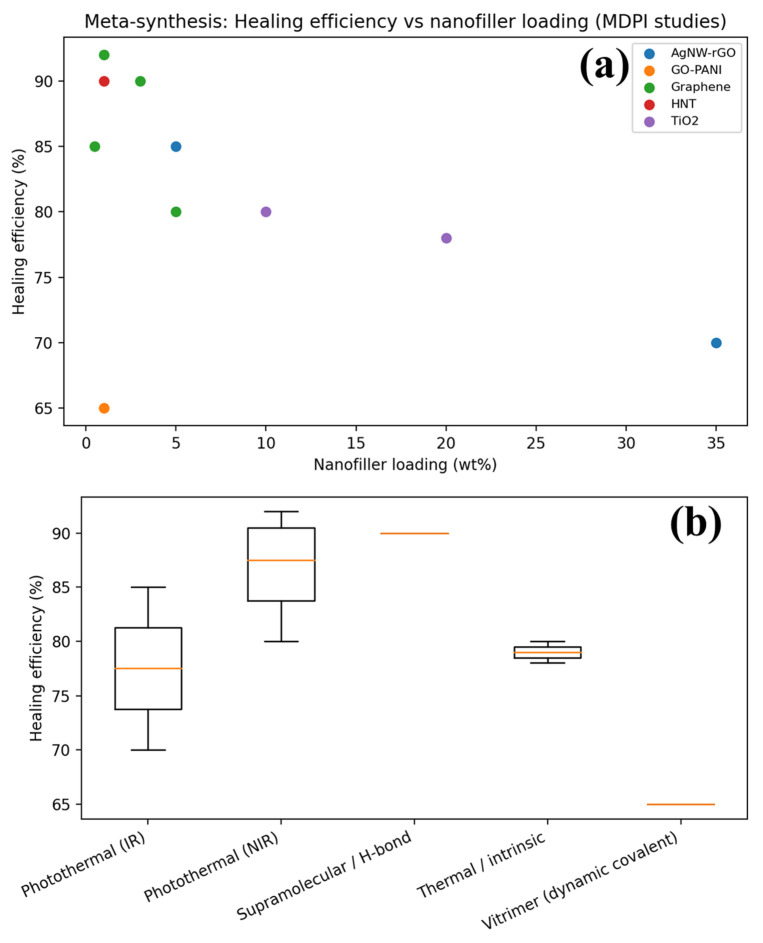
(**a**,**b**) Meta-analysis of healing efficiency (η) versus nanofiller loading (wt%) for representative self-healing polymer nanocomposites, (**b**) Mechanism-wise distribution of reported healing efficiencies (η) for self-healing polymer nanocomposites. Data extracted/compiled from [74,97,98,99,100].

To address the limited comparability of qualitative claims across studies, a cross-study quantitative meta-synthesis was performed by extracting reported healing efficiency (η) values and nanofiller loading levels [74,97,98,99,100]. The resulting compilation provides a literature-grounded benchmark for identifying practical filler loading windows and understanding why conflicting healing trends are frequently reported. As shown in Figure 4a, healing efficiency does not increase monotonically with nanofiller loading. Instead, most systems exhibit an optimum healing window at intermediate filler contents, where stimulus transduction or reinforcement is improved, while polymer mobility remains sufficiently high for chain diffusion and/or dynamic bond exchange [98,99]. At higher filler contents, η commonly decrease due to nanoscale confinement, interphase immobilization, and crack-tip stress localization [100]. This supports the critical design rule that overloading nanofillers can suppress healing even when modulus and conductivity increase, directly explaining contradictions reported across the literature.

Mechanism-specific trends are further summarized in Figure 4b, which presents the distribution of healing efficiencies grouped by healing strategy (photothermal, thermal/intrinsic, supramolecular, dynamic covalent/vitrimer) [99]. Although high η values are reported for several mechanisms, the distributions highlight that performance is strongly dependent on activation conditions and nanocomposite architecture. Importantly, the spread in η reinforces that single-study or single-cycle results cannot be treated as universal indicators, and that quantitative benchmarking requires standardized reporting of damage geometry, healing conditions, and multi-cycle durability [98,99,100]. Collectively, Figure 4a,b provide evidence-based guidance for selecting filler loading regimes and mechanism classes suitable for targeted applications.

Overall, these quantitative ranges indicate that optimal self-healing nanocomposite performance is typically achieved within narrow filler loading windows, often near functional thresholds such as percolation. However, exceeding these regimes can amplify interphase immobilization and network confinement, reducing chain mobility and multi-cycle healing retention. This quantitative basis directly leads to the strength–healing trade-off in nanocomposites, which is critically synthesized in Section 4.2.

### 4.2. Strength–Healing Trade-Off in Self-Healing Polymer Nanocomposites: Critical Synthesis and Design Boundaries

Self-healing polymer nanocomposites are governed by competing requirements between mechanical reinforcement and intrinsic healability. High stiffness, strength, and dimensional stability are generally obtained through dense crosslinking, rigid reinforcement phases, and filler network formation, all of which restrict polymer segmental mobility [101]. In contrast, intrinsic self-healing typically requires sufficient chain mobility at the damaged interface to promote crack-plane wetting, segmental interdiffusion, and/or reversible bond exchange [102]. Consequently, many studies report a strength–healing trade-off, where improvements in modulus and strength are accompanied by reduced healing kinetics, decreased healing efficiency, and weakened repeatability under multi-cycle damage–healing conditions [101,102,103]. Importantly, this trade-off should not be interpreted as a universal limitation; instead, it reflects a tunable design space governed by the interplay between nanofiller geometry, dispersion quality, surface chemistry, polymer network architecture, and healing activation pathway.

**Figure 5:**
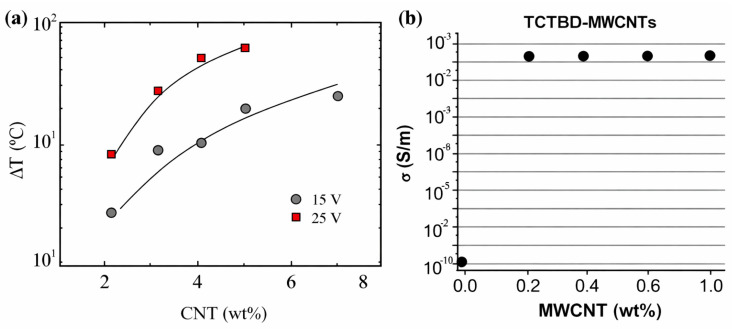
Percolation-controlled transport response in CNT nanocomposites: implications for the strength−healing trade-off indicating (**a**) Joule heating response (ΔT vs. CNT wt%) [109], and (**b**) Electrical percolation behaviour (σ vs. CNT wt%) [110].

At the molecular scale, intrinsic healing is enabled by mechanisms such as polymer chain interpenetration across crack faces, relaxation-driven crack closure, and dynamic reformation of reversible interactions (e.g., supramolecular bonding, Diels–Alder reactions, disulfide exchange, or transesterification in vitrimers) [104]. All these processes rely on polymer segments near the crack plane remaining sufficiently mobile over relevant timescales. However, increased crosslink density reduces free volume and segmental relaxation while simultaneously increasing the activation barrier for network rearrangement. As a result, healing becomes slower and more incomplete unless adequate stimulus energy is supplied (thermal, electrical, photothermal, or solvent-assisted), and the recovered mechanical performance may decline in repeated cycles due to partial bond exhaustion or irreversible structural rearrangement [105]. In nanocomposites, this mobility suppression is amplified by the formation of a filler–polymer interphase region. Near nanofiller surfaces, polymer chains can become immobilized due to adsorption, hydrogen bonding, π–π interactions, covalent grafting, or confinement-induced crystallization [106]. Because the interphase volume fraction increases rapidly with nanofiller surface area and loading, even modest filler contents can restrict mobility significantly, especially when fillers possess high surface energy or strong chemical functionality. This explains why certain nanofiller additions produce substantial mechanical reinforcement while simultaneously suppressing intrinsic healing. However, the existence of an optimum nanofiller window and the percolation-driven transition is clearly evidenced in conductive CNT-based nanocomposites [107]. In such systems, a small increase in CNT loading near the percolation threshold results in a disproportionate rise in electrical conductivity and Joule heating efficiency, enabling rapid and localized temperature elevation that can trigger dynamic bond exchange and accelerate intrinsic healing [108]. However, once a dense percolated network is established, further CNT addition progressively increases interphase immobilization and confinement, which suppresses segmental mobility and reduces multi-cycle healing retention. This transition from transport-assisted healing enhancement to mobility-restricted healing suppression is central to the reinforcement–healability contradiction in self-healing nanocomposites [109,110]. This percolation-controlled functional transition is illustrated in Figure 5a, which combines representative data on Joule heating response and electrical percolation behavior as a function of CNT content. As shown in Figure 5a, ΔT increases sharply with CNT loading under applied voltage, confirming percolation-controlled Joule heating [109]. Figure 5b similarly shows an abrupt conductivity rise once the percolation threshold is reached [110]. These trends highlight an optimum filler window—percolation enhances stimulus-driven healing, whereas excessive loading restricts mobility and reduces healing repeatability.

A defining contradiction in self-healing nanocomposites is that nanofillers may enhance healing kinetics through transport-related effects while simultaneously suppressing healing through confinement and percolation-induced stiffening. Conductive fillers such as carbon nanotubes, graphene derivatives, MXenes, and metallic nanowires are often introduced to enable stimulus transduction, particularly Joule heating or photothermal heating, which can accelerate dynamic bond exchange in intrinsic networks [111]. Percolated conductive pathways provide rapid and localized thermal delivery, improving healing rate and allowing healing to be activated without global heating [112]. However, once filler loading exceeds the percolation threshold, filler networks frequently become mechanically constrained. Polymer segments are confined within the network, the immobilized interphase volume grows, and crack-plane diffusion becomes increasingly hindered [113]. Therefore, healing efficiency often exhibits a non-monotonic dependence on filler loading: at low loadings, reinforcement is limited and healing is dominated by polymer chemistry; at intermediate loadings near percolation, healing may improve due to crack bridging, enhanced energy dissipation, and improved heat transport; while at higher loadings, healing declines because mobility suppression becomes dominant. This percolation–mobility conflict provides a mechanistic basis for contradictory literature outcomes in which the same filler is reported to either improve or reduce healing performance, since dispersion quality, filler functionalization, polymer Tg relative to healing temperature, and testing protocols significantly affect whether transport enhancement outweighs mobility restriction [114].

From a design standpoint, the strength–healing trade-off can be rationalized through a set of quantitative control variables that collectively define the feasible design space. Crosslink density influences stiffness and activation barriers for bond exchange. Filler loading and filler aspect ratio control reinforcement, crack bridging, and the interphase fraction. The percolation threshold governs whether conductive or thermal networks can be formed at low loading; and, critically, interphase mobility determines whether the interface behaves as a rigid immobilized region or a dynamic, healing-active layer [115]. When interfacial bonding is permanent and highly restrictive, reinforcement is enhanced but healing deteriorates sharply. In contrast, when the filler surface is engineered with reversible chemistry such as dynamic covalent linkages or supramolecular anchoring stress transfer can be maintained while allowing for molecular rearrangement required for healing [116]. This highlights that the optimal design is not necessarily the maximum filler loading or maximum crosslink density, but rather a balanced configuration where transport-assisted activation and dynamic interphase interactions compensate for mobility constraints.

Achieving simultaneous reinforcement and healability requires deliberate strategies that shift the balance away from permanent confinement and toward reversible interphase dynamics. Interphase engineering, in which filler surfaces are functionalized to promote reversible interactions, is among the most effective approaches to mitigate the trade-off, as it reduces irreversible immobilization while improving crack bridging and stress redistribution [117]. Similarly, hierarchical filler architectures and hybrid filler systems can deliver reinforcement and stimulus functionality at reduced loading, limiting the immobilized interphase fraction while retaining multifunctionality [66]. Stimulus localization represents another key strategy: conductive networks can be designed to deliver localized heat to damaged zones, accelerating bond exchange without globally softening the polymer, thereby improving healing while maintaining structural stability [118]. Additionally, matching polymer Tg and service temperature conditions can improve healability without sacrificing modulus excessively, particularly when healing is dominated by segmental diffusion or supramolecular rearrangement.

Finally, the strength–healing trade-off must be evaluated using engineering-relevant metrics rather than single-cycle recovery values alone. Many studies reports healing efficiency as recovery in tensile strength or strain-to-failure after a single cut-and-heal occurrence; however, this approach can be misleading because the results are strongly affected by viscoelastic effects, specimen geometry, strain rate, and crack sharpness [119,120,121]. For practical relevance, self-healing nanocomposites should demonstrate stable multi-cycle recovery and improved resistance to progressive damage under fatigue. These inconsistencies collectively indicate that reported synergistic improvements in self-healing nanocomposites are highly condition-dependent rather than universally reproducible. In many systems, the dominant mechanism shifts with filler dispersion quality, interfacial chemistry, and the activation temperature relative to Tg, which controls whether healing is governed mainly by chain diffusion, dynamic bond exchange, or interfacial rebonding [8,122]. Moreover, variations in damage geometry, healing time, and cycle number can produce substantially different recovery values even for comparable formulations. Therefore, fracture-based metrics (recovery in fracture toughness, crack growth suppression), multi-cycle retention (performance after repeated healing cycles), and durability under environmental aging (humidity, UV, thermal exposure) remains critical. Overall, this critical synthesis shows that the strength–healing trade-off in nanocomposites emerges from competition between mobility restriction (crosslinking and interphase confinement) and activation enhancement (percolation-enabled stimulus transduction) [32]. By engineering dynamic interfaces and optimizing filler contents near functional thresholds without excessive confinement, self-healing polymer nanocomposites can be designed to balance stiffness, multifunctionality, and repeatable healing under realistic service conditions.

### 4.3. Engineering Performance Requirements for Structural Relevance

Although self-healing performance is commonly reported as a percentage recovery in tensile strength or elongation after a single damage–heal event, such metrics alone are insufficient to establish engineering relevance. In structural service conditions, polymeric components rarely fail due to a single catastrophic cut; instead, damage accumulates progressively through microcracking, delamination, fatigue loading, and environmental aging [5]. Therefore, structurally meaningful self-healing should be evaluated based on the ability to suppress crack growth and to retain mechanical integrity under repeated loading/healing cycles rather than achieving maximum single-cycle recovery. In this context, the minimum requirement for structural deployment is not merely high initial healing efficiency, but stable multi-cycle retention, resistance to fatigue-driven crack propagation, and tolerance to realistic environmental exposures (humidity, UV, thermal cycling, and chemicals) [7]. Importantly, systems that show high one-time recovery may still fail under cyclic conditions if filler networks, interfaces, or dynamic bonds undergo irreversible rearrangement or progressive degradation [123].

A practical engineering translation can be expressed through performance thresholds: (i) the healed structure should recover sufficient stiffness/strength to prevent rapid stiffness drop or instability; (ii) healing should remain functional over multiple damage–healing cycles (typically ≥3–10 cycles for meaningful reliability demonstration); and (iii) healing should remain effective under fatigue loading, where microcracks continuously form and propagate [34]. Consequently, fracture-based metrics such as recovery of interfacial fracture toughness, reduction in fatigue crack growth rate, and restoration of load transfer across damaged interfaces are more representative than single tensile recovery [124]. Environmental durability is equally essential, since moisture uptake, oxidation, UV exposure, and thermal aging can alter interphase chemistry, disrupt supramolecular interactions, and decrease the kinetics of dynamic bond exchange, leading to reduced healing repeatability [125]. Therefore, the design objective should shift from maximizing single-event healing efficiency to maximizing damage tolerance, fatigue life extension, and multi-cycle recovery stability, which represent the critical constraints for structural applications.

**Figure 6:**
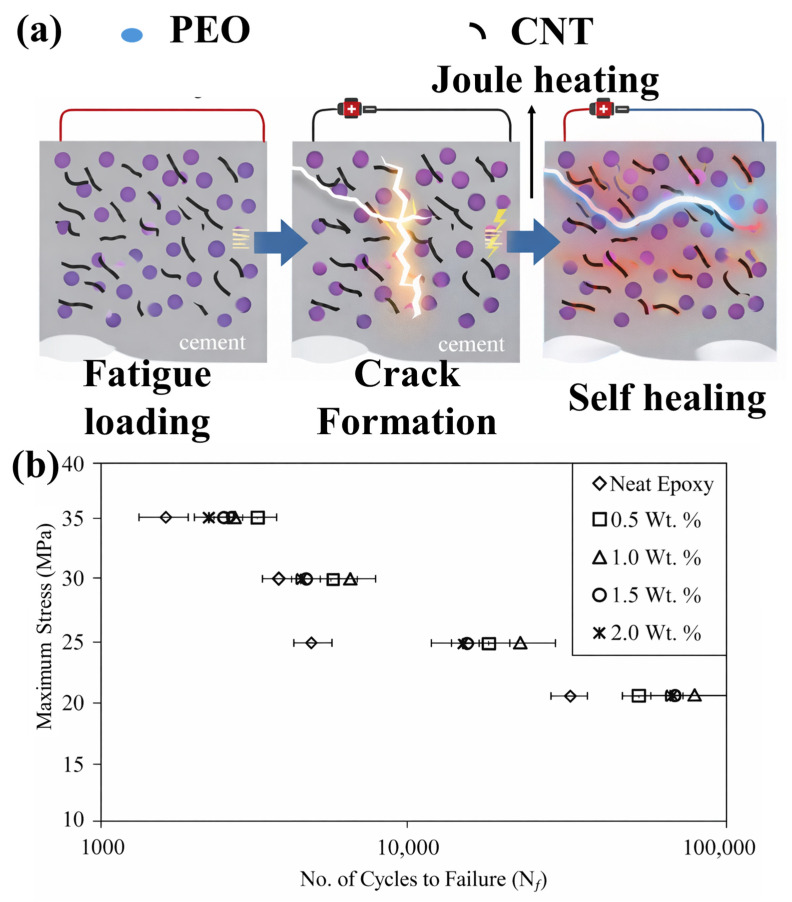
Engineering-relevant durability and multi-cycle healing in self-healing polymer nanocomposites. (**a**) Schematic illustration of fatigue damage accumulation and stimulus-assisted healing under cyclic loading in nanofiller-reinforced polymer networks. (**b**) Representative recovery trends after repeated damage–healing cycles, showing retention of mechanical performance and fatigue life extension under optimized nanofiller loading and activation conditions [126].

The importance of fatigue-driven performance and repeatable healing is illustrated in Figure 6a, where conductivity-enabled Joule heating is used to activate repair after cyclic damage, delaying rapid failure and extending fatigue life under repeated loading [126]. This evidence reinforces that structural relevance demands healing concepts that are compatible with fatigue accumulation and that can be externally or autonomously triggered without compromising load-bearing stability. In Figure 6b, the fatigue response is presented as maximum stress versus cycles to failure, clearly demonstrating fatigue-life improvement in microcapsule-modified epoxy compared with neat epoxy [126]. At comparable stress levels, the self-healing formulations sustain a higher number of cycles before failure, confirming that healing strategies can effectively delay crack propagation and extend durability under cyclic loading.

#### Standardization of Healing Metrics and Test Protocols in Polymer Nanocomposites

A major barrier to translating self-healing polymer nanocomposites into engineering practice is the lack of standardized testing protocols, which makes cross-study comparison unreliable. Reported healing efficiency (η) varies widely not only due to intrinsic material chemistry and nanofiller design, but also because of differences in damage geometry, specimen size, strain rate, healing conditions, and evaluation metric [100,127]. Consequently, high healing efficiency values often reflect favorable test conditions rather than robust material performance [128]. Tensile-based healing metrics, although widely used, can be misleading when treated as a universal indicator of healing. Many studies report η as recovery of ultimate tensile strength or elongation after a single cut-and-heal outcome [128,129,130,131,132]. However, tensile recovery is strongly influenced by viscoelastic effects, plastic deformation, crack closure behavior, and stress redistribution by fillers, and does not necessarily represent recovery of crack resistance [132]. In nanocomposites specifically, reinforcement can artificially improve tensile response through crack-bridging and load transfer even if true interfacial re-bonding and fracture resistance remain limited [133]. Therefore, tensile recovery alone can overestimate healing and fails to predict performance under cyclic loading.

For structural relevance, fracture- and fatigue-based metrics must be emphasized because real components fail through crack initiation and growth rather than monotonic tensile rupture. Recommended measures include recovery of fracture toughness, suppression of crack growth rate under fatigue (da/dN vs. ΔK), and retention of mechanical properties after multiple healing cycles [134]. Importantly, healing should be evaluated using multi-cycle retention, since many nanocomposites show decreased healing in subsequent cycles due to interphase immobilization, filler rearrangement, and progressive bond exhaustion [135]. Hence, single cycle η should be reported only as an initial screening metric rather than as proof of durability. To improve reproducibility and enable meaningful comparison across systems, minimum reporting standards should be adopted for self-healing polymer nanocomposites, including damage definition, healing conditions, and metric selection. A practical standardized checklist is summarized in Table 6.

Standardized reporting and fracture/fatigue-based evaluation are therefore essential for establishing true structure–property–healing relationships and for preventing misleading comparisons between nanocomposite systems. Adoption of common protocols will significantly accelerate engineering translation and meaningful benchmarking of next-generation self-healing polymer nanocomposites.

## 5. Stimuli-Responsive Self-Healing Polymer Nanocomposites

Stimuli-responsive self-healing polymer nanocomposites enable damage repair to be triggered or accelerated by external inputs such as heat, light, electric fields, or magnetic fields [127]. In these systems, nanofillers are not passive reinforcements; rather, they act as stimulus transducers that convert external energy into localized thermal or physicochemical activation capable of initiating bond exchange, interdiffusion, or network rearrangement [149]. Compared with autonomous/passive healing, stimuli-responsive platforms offer control over healing rate, location, and activation conditions, which is essential for coatings, electronics, and structurally loaded parts [150].

Thermally activated healing remains widely adopted because most intrinsic healing chemistries—including reversible covalent exchange and diffusion-driven welding—exhibit accelerated kinetics at elevated temperatures [151,152]. Incorporating high-thermal-conductivity fillers such as graphene, carbon nanotubes, and selected metal oxides improves heat distribution, minimizing thermal gradients and enabling more uniform healing in thick or complex geometries [153]. However, thermal pathways must be balanced against filler-induced chain immobilization at the interphase, which can reduce mobility-dependent healing if filler content becomes excessive [154,155].

Photothermal activation provides spatially selective healing by exploiting fillers that absorb visible/IR irradiation and convert it to heat through non-radiative relaxation [156]. When dispersed within self-healing polymers, these nanofillers enable remote, localized heating at damage sites without globally raising the component temperature, which is particularly advantageous for coatings, flexible electronics, and thermally sensitive devices [99]. The achievable healing rate depends strongly on optical absorption efficiency, filler dispersion, and interfacial thermal transport [157].

**Figure 7:**
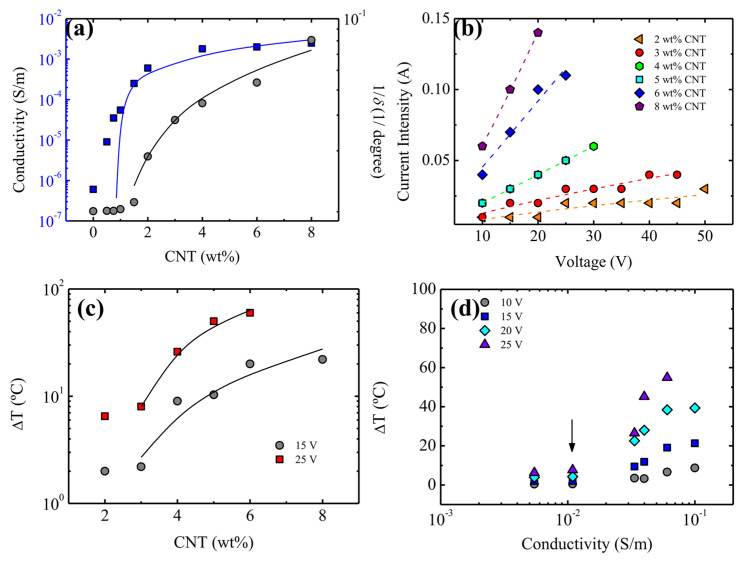
(**a**) Electrical conductivity as a function of CNT loading, showing percolation−driven conductivity increase, (**b**) Current intensity versus applied voltage for different CNT loadings, (**c**) Temperature rise (ΔT) as a function of CNT concentration at selected voltages, and (**d**) Correlation between ΔT and conductivity, confirming that effective Joule heating is achieved only beyond the percolation regime [109].

Electrically triggered healing is especially attractive for rapid and programmable repair. Here, conductive fillers form percolated networks that allow for current flow, generating local Joule heating and activating intrinsic healing (e.g., dynamic exchange or mobility enhancement) in the damaged region [158,159,160]. The quantitative importance of percolation is illustrated in Figure 7a–d indicating conductivity rises sharply once CNT loading reaches the percolation regime (Figure 7a), which increases current intensity at a given voltage (Figure 7b) and produces a pronounced temperature rise (ΔT) (Figure 7c). The correlation between ΔT and conductivity (Figure 7d) confirms that effective electrical activation requires conductivity above the percolation threshold, making filler loading a key design variable [109]. At the same time, high filler contents that promote conductivity can also restrict polymer segmental motion, highlighting a fundamental activation–mobility trade-off in electrically healable nanocomposites [109,161].

Magnetically triggered healing offers wireless activation in nanocomposites containing magnetic nanoparticles. Under alternating magnetic fields, heat is generated through hysteresis loss or Néel/Brownian relaxation, enabling localized repair in enclosed or embedded components where optical/electrical access is limited [135,162]. Although less widely reported than electrical or photothermal strategies, magnetic approaches provide unique integration opportunities for sealed structures and remote-healing architectures [163]. Environmental/chemical stimuli (e.g., moisture, pH, solvents) can also promote healing by plasticization or reversible interfacial interactions; nanofillers can amplify sensitivity through increased surface area and localized chemical microenvironments [164,165].

Across all stimuli-responsive strategies, performance is governed by nanofiller dispersion, percolation threshold, and interfacial compatibility. Continuous and uniform networks improve stimulus transduction efficiency, whereas excessive loading can suppress healing by mobility restriction and interphase confinement [166]. In addition, repeated stimulus cycling may induce filler rearrangement or interfacial degradation, emphasizing the need for durability-focused metrics beyond single-cycle healing [167]. Overall, nanofiller-assisted stimulus transduction is central to improving healing controllability while simultaneously enabling multifunctionality (e.g., conductivity, sensing, thermal management), which supports real-time damage monitoring and programmable repair in smart material platforms [168]. A concise overview of the main stimuli-responsive routes and their activation mechanisms is summarized in Table 7.

Overall, the strategic integration of external stimuli such as thermal, electrical, optical, or magnetic inputs with nanofiller-enabled transduction pathways constitutes a pivotal advancement toward the practical realization of self-healing polymer nanocomposites. By enabling efficient stimulus absorption, localized energy conversion, and targeted activation of healing mechanisms, these integrated systems significantly enhance healing efficiency, spatial control, and functional reliability under real-world operating conditions. Such stimulus-responsive, nanofiller-assisted healing strategies not only broaden the applicability of self-healing materials but also accelerate their transition from laboratory-scale concepts to robust, high-performance technologies for advanced functional applications. These developments and their associated design principles are discussed in detail in the following section.

## 6. Emerging Applications and Deployment Readiness of Self-Healing Polymer Nanocomposites

While self-healing polymer nanocomposites have been proposed for a wide range of functional and structural applications, their transition to real-world deployment is primarily governed by whether they can meet quantified engineering benchmarks under realistic service conditions. In contrast to laboratory demonstrations that often focus on single-cycle tensile recovery, practical applications demand multi-cycle durability, long-term stability, and performance retention under cyclic loading, thermal cycling, humidity exposure, and abrasion. Therefore, the relevance of each application domain is best evaluated using a technology readiness and performance-gap perspective, rather than descriptive enumeration. From a deployment standpoint, applications can be broadly classified into: (i) near-term, performance-tolerant systems (coatings, adhesives, wearable electronics), where partial property recovery may still deliver functional value; (ii) mid-term opportunities (structural composites for transportation), where healing must occur without sacrificing stiffness, strength, and fatigue resistance; and (iii) long-term targets (aerospace-grade composites), where stringent certification requirements and reliability constraints impose substantial barriers [174,175,176]. In conductive and stimulus-responsive systems, nanofillers additionally enable multifunctionality such as electrical sensing and Joule/photothermal activation, but these advantages must be balanced against filler-induced mobility restriction and long-term interfacial degradation [177]. To clarify realistic translation pathways, Table 8 summarizes the principal application areas, their target benchmarks, the current best reported performance trends, and the remaining quantified gaps that must be bridged for commercial relevance. Importantly, across most sectors, the dominant limitation is not the ability to trigger healing, but achieving simultaneously high mechanical performance, repeatable healing retention, and durability under fatigue and environmental aging [178].

Overall, the most deployment-ready applications are coatings, sealants, adhesives, and flexible electronics, where functional recovery can deliver immediate value even if healing is incomplete [186]. In contrast, load-bearing structural composites remain constrained by the intrinsic strength–healing trade-off, particularly under fatigue loading where crack growth suppression must be demonstrated using standardized fracture-based metrics [187]. However, the identification of performance gaps will require nanocomposite architectures that preserve mobility (e.g., dynamic interphase engineering, gradient filler distribution) while maintaining reinforcement efficiency and long-term durability under cyclic service conditions [188].

## 7. Challenges, Limitations, and Technological Barriers

Despite substantial progress in the development of self-healing polymer nanocomposites, several fundamental and practical barriers continue to limit their translation into real-world technologies. The central challenge lies in achieving simultaneous mechanical reliability, repeatable healing, multifunctionality, and long-term durability within scalable material architectures [189]. While the strength–healing contradiction arising from mobility restriction versus reinforcement is critically discussed in Section 4.2 emphasizes the remaining constraints associated with processing scalability, durability under service conditions, and standardization of evaluation protocols, which currently hinder meaningful benchmarking and industrial adoption. A persistent materials-level limitation arises from nanofiller dispersion and interfacial compatibility. Uniform dispersion is difficult to achieve at industrially relevant scales, particularly for high-aspect-ratio fillers prone to agglomeration, which can introduce stress concentrators and cause variability in both mechanical and healing performance [190]. Moreover, interfacial interactions must be carefully tuned: excessively strong filler–polymer bonding can immobilize chains near the interphase and suppress healing kinetics, whereas weak interactions compromise stress transfer and reinforcement [191]. Thus, designing interphases with dynamic and reversible interactions remains essential to balance durability, reinforcement, and recoverability.

Durability under realistic operating environments represents a major technological bottleneck. Many studies evaluate healing over a limited number of damage–healing cycles under controlled laboratory conditions, whereas practical components are subjected to complex stress states, cyclic loading, thermal fluctuations, humidity, ultraviolet radiation, and chemical exposure [192,193]. These factors can degrade dynamic networks and weaken interfacial integrity, leading to progressive decline in healing retention. Therefore, systematic durability assessment under fatigue and environmental aging remains insufficient and should be incorporated into future benchmarking studies [194]. Scalability and manufacturing compatibility further constrain translation. Several reported systems require specialized synthesis pathways, surface functionalization procedures, or processing conditions that are difficult to integrate into high-throughput manufacturing [195]. In addition, achieving consistent nanofiller dispersion and percolated architecture during conventional polymer processing remains challenging. Development of scalable and processing-compatible fabrication routes that retain controlled interphase design is therefore a key priority [196].

Finally, the lack of standardization in evaluation protocols limits reproducibility and slows technology transfer. Healing performance is reported using different damage geometries, healing conditions, and metrics, making direct comparison across studies unreliable. Establishing unified testing methodologies, reporting requirements, and benchmarking criteria would significantly strengthen the field and accelerate the selection of nanocomposite systems for specific engineering applications [197]. Overall, overcoming these interconnected barriers will require coordinated advances in polymer chemistry, nanofiller engineering, scalable processing, and application-driven durability testing.

### 7.1. Innovation Pathways and Roadmap for Next-Generation Self-Healing Polymer Nanocomposites

To move beyond proof-of-concept demonstrations and enable practical deployment, future research must shift from incremental improvements in healing efficiency toward architecture- and data-driven design strategies that explicitly resolve the competing requirements of reinforcement, mobility, and durability. Several innovation pathways are particularly promising for establishing reproducible performance gains and reducing the strength–healing contradiction discussed throughout this review.

1. *Dynamic interphase engineering:* Rather than treating the filler–matrix interface as a static adhesion zone, emerging approaches design the interphase as an active healing region by incorporating reversible covalent chemistry, supramolecular motifs, or exchangeable grafted chains at the nanofiller surface [198]. This dynamic interphase concept enables strong load transfer during service while preserving localized mobility and bond exchange near crack surfaces, improving multi-cycle healing retention without sacrificing modulus. Interphase design should be quantified using interphase thickness, Tg shifts near fillers, and interfacial exchange kinetics to establish transferable design rules [199].
2. *Gradient and hierarchical filler architectures:* Uniform nanofiller loading across the bulk often forces a compromise between percolation-enabled functionality and mobility-dependent healing. A more effective pathway is spatially programmed architectures such as gradient distributions (filler-rich layers for conductivity/sensing, filler-lean healing zones for mobility), crack-tip targeted reinforcements, or hierarchical hybrid networks (e.g., 1D CNT + 2D graphene) that reduce percolation threshold while minimizing confinement [200,201]. Such architecture can decouple stiffness and healing by localizing reinforcement and stimulus transduction away from regions where chain diffusion is required.
3. *Machine learning and data-driven optimization of design windows:* The nanocomposite design space is intrinsically high-dimensional (polymer chemistry, dynamic bond density, filler geometry/aspect ratio, interphase chemistry, percolation threshold, processing route, stimulus conditions). Machine learning (ML) and Bayesian optimization can accelerate the identification of quantitative design windows by learning structure–property–healing mappings from curated datasets [202]. Integrating ML with experimentally validated descriptors (e.g., φc, AR, interphase mobility indices, Tg shifts, conductivity–ΔT relationships) provides a realistic pathway for predictive formulation and reduced trial-and-error iteration [203].
4. *Reliability-focused testing and standardization as an innovation enabler:* Finally, translation requires not only new materials but also credible qualification frameworks. Future work should prioritize fracture- and fatigue-based healing metrics, multi-cycle durability benchmarking, and accelerated aging protocols (UV, humidity, thermal oxidation, cyclic fatigue) [204]. Adoption of standardized reporting and minimum test protocols will enable meta-analysis, cross-laboratory reproducibility, and rational comparison of competing nanofiller strategies. Coupling standardized testing with digital datasets further strengthens ML-driven design and supports application-driven certification pathways [205].

Overall, these innovative routes dynamic interphase design, spatially programmed nanofiller architectures, data-driven optimization, and reliability-centered qualification define a practical roadmap for converting self-healing nanocomposites from laboratory-scale demonstrations into scalable and application-validated engineering materials.

### 7.2. Degradation Catalysis, Nanofiller Release Risk, Toxicity, and Cost–Performance Trade-Offs

Despite rapid progress in functional and structural self-healing polymer nanocomposites, their practical translation is constrained by risks that are often under-discussed in the self-healing literature, namely nanofiller-assisted degradation, nanoparticle release and environmental fate, and cost–performance justification relative to non-healing high-performance composites [206]. These factors become particularly critical for long-life structural applications and consumer-facing products, where regulatory compliance, stability under aging, and lifecycle impacts must be demonstrated. First, several nanofillers can influence polymer aging through unintended catalytic or photochemical pathways. Metal and metal-oxide nanoparticles may accelerate oxidation or hydrolysis by promoting radical formation, altering local oxygen diffusion, or acting as catalytic sites, potentially reducing long-term ductility and suppressing multi-cycle healing [207]. Similarly, carbon-based fillers can modify thermal history and stress localization, which may accelerate microcrack formation under cyclic loading [208]. Therefore, healing metrics reported at short timescales (hours–days) should be complemented by aging-aware evaluation, including thermal–oxidative aging, UV exposure (for coatings), and long-term cyclic fatigue protocols [135,209]. Secondly, environmental and health considerations are increasingly relevant. Under abrasion, fatigue crack propagation, or weathering, nanocomposites may undergo filler debonding, interphase fragmentation, or particle liberation, raising concerns related to nanoparticle inhalation risk, aquatic contamination, and end-of-life management [210,211]. This is particularly relevant for graphene/CNT systems and oxide nanoparticle fillers. Lifecycle safety assessment should therefore include filler containment strategies (e.g., strong interfacial anchoring, encapsulated fillers, crosslinked barrier layers), alongside appropriate regulatory compliance documentation [212]. Thirdly, the cost–performance balance must be explicitly considered. While nanofillers can impart multifunctionality and enable fast stimulus-triggered healing, high-quality conductive fillers (e.g., CNTs, graphene derivatives, MXenes) can significantly increase formulation cost and processing complexity [213]. For some applications, non-healing high-performance composites may still provide a lower-cost route to reliability through damage tolerance, protective coatings, and scheduled maintenance [214]. Thus, adoption of self-healing nanocomposites will be strongest where healing offers unique value (inaccessible repair zones, real-time sensing and healing, fatigue crack suppression, reduced downtime), rather than as a generalized replacement of conventional composites. To support safe and scalable translation, future work should integrate accelerated aging studies, nanofiller-release characterization, toxicity screening, and cost–benefit assessment into the standard development pathway for self-healing nanocomposites [215]. A concise risk–mitigation overview for common nanofiller classes is provided in Table 9.

## 8. Conclusions and Future Perspectives

Self-healing polymer nanocomposites represent a decisive step beyond conventional self-healing polymers by enabling simultaneous durability recovery and functional reinforcement within a single material platform. Rather than acting only as passive strengthening agents, nanofillers actively regulate healing by modifying crack evolution, stress redistribution, interfacial dynamics, and stimulus transduction. Across intrinsic and extrinsic architectures, this review establishes that the most consistent pathway toward high-performance systems is not maximizing either healing efficiency or stiffness independently, but engineering structure–property–healing coupling so that reinforcement does not eliminate the molecular mobility required for repair. In this context, the central scientific contribution of this review is the consolidation of evidence showing that nanocomposite performance is governed by a constrained design window defined by competing parameters—filler loading, aspect ratio, dispersion quality, interphase confinement, and activation conditions—rather than by isolated chemical mechanisms.

A key conclusion is that intrinsic healing systems (dynamic covalent networks, supramolecular bonding, and chain diffusion) provide the best foundation for repeated healing, but only when nanofiller–polymer interphases are tuned to remain dynamic and not permanently immobilizing. Conversely, extrinsic systems (microcapsules and vascular architectures) can enable autonomous single-event repair with high local effectiveness, but are intrinsically limited by agent depletion, crack path dependence, and processing complexity. Stimuli-responsive nanocomposite systems (Joule heating, photothermal, magnetic, and humidity/solvent-triggered healing) further expand functionality by enabling spatially localized, on-demand healing; however, their long-term effectiveness depends strongly on percolation stability, interfacial fatigue resistance, and the ability to maintain conductive networks under cyclic damage. Importantly, engineering translation requires a shift in evaluation metrics. Much of the literature still reports healing as single-cycle tensile recovery, which can overestimate true durability. For structural or fatigue-loaded applications, the more relevant targets include fracture toughness recovery, fatigue crack growth resistance, cyclic healing retention, and environmental aging stability, since service failure is typically governed by crack propagation under complex loading rather than monotonic failure. Therefore, future progress will depend as much on standardization of testing protocols as on advances in chemistry and nanofiller design. Standardized reporting of damage geometry, healing activation conditions, number of cycles, and recovery metrics will allow for cross-study comparison and will accelerate technology readiness assessment. Design principles distilled from this review: (i) optimal self-healing nanocomposites are typically achieved within a narrow filler-loading window near functional thresholds (e.g., electrical percolation), where transport-assisted activation maximizes healing kinetics without severe confinement; (ii) dynamic interphase engineering (reversible covalent or supramolecular interfacial bonding) is the most effective strategy to decouple reinforcement from healability; (iii) engineering relevance requires multi-cycle and fatigue-informed recovery metrics rather than single-event tensile recovery; and (iv) translation readiness must incorporate environmental aging, nanofiller release risk, and scalable processing constraints

Looking forward, the most promising research directions include (i) hierarchical or spatially programmed architectures where highly mobile healing zones coexist with reinforced load-bearing domains, (ii) vitrimer and covalent adaptable networks integrated with nanofillers to provide reprocessability, healability, and toughness simultaneously, (iii) closed-loop intelligent materials combining damage sensing, autonomous decision-making, and healing actuation, and (iv) data-driven optimization (multiscale modeling and machine learning) to navigate the high-dimensional design space defined by polymer chemistry, nanofiller geometry, dispersion, and processing. Ultimately, self-healing polymer nanocomposites will achieve broad adoption only when they demonstrate predictable performance under realistic service conditions, quantified trade-off boundaries, and manufacturing-ready scalability—transforming self-healing from a laboratory concept into a deployable engineering technology.

## Figures and Tables

**Table 1:** Provides a comparative overview of the review landscape and explicitly shows the additional insight delivered by the present work.

Researcher	Main Scope of Review	Covers Nanofillers	Covers Interphase/Percolation Analysis	Quantitative Synthesis	Engineering Translation (Fatigue/Multicycle/Environment)	Key Limitation	Present Review Advantage
Hornat et al. [17]	Intrinsic self-healing polymers (dynamic covalent)	Partial	Limited	No	Limited	Chemistry-focused; nanofiller role weak	Adds nanofiller–healing coupling + design rules
Kanu et al. [18]	Extrinsic systems (capsules/vascular)	No	No	No	Partial	Not nanocomposite-centric	Dedicated nanocomposite interphase/percolation
Orellana et al. [19]	Joule-healing conductive composites	Yes	Partial	Limited	Limited	Mostly application summary	Adds percolation–mobility conflict + quant ranges
Zhou et al. [20]	Supramolecular self-healing systems	Partial	No	Limited	No	Overly descriptive	Adds quantitative synthesis + contradictions
Schenk et al. [21]	Vitrimer composites	Yes	Partial	Limited	Partial	Narrow to vitrimers	Full landscape + cross-mechanism comparison
Van et al. [22]	Multifunctional self-healing composites	Yes	Limited	No	Partial	No protocol discussion	Adds standardization roadmap
Irzhak et al. [23]	Self-healing nanocomposites general	Yes	Limited	Limited	Limited	Categorization; lacks design boundaries	Adds design boundaries + engineering thresholds
Parihar et al. [24]	Nanofiller reinforcement effects	Yes	Yes	Limited	No	Reinforcement focus; healing secondary	Full structure–property–healing integration
Present review	Self-healing polymer nanocomposites: critical synthesis	Yes	Yes (interphase + percolation)	Yes (design ranges & trends)	Yes (fatigue & multicycle durability)	—	Mechanism-driven contradictions + actionable design principles

**Table 2:** Analytical comparison of intrinsic and extrinsic self-healing mechanisms in polymer systems, highlighting triggers, repeatability, and kinetics.

Comparison Criterion	Extrinsic Self-Healing (Capsules/Vascular)	Intrinsic Self-Healing (Dynamic/Supramolecular Networks)
Healing principle [36]	Stored healing agent released into crack plane	Reversible bond reformation/bond exchange within network
Repeatability [37]	Limited (one-time or few cycles); depends on reservoir	High; multi-cycle feasible if network remains active
Healing kinetics [38]	Often fast (agent flow + polymerization)	Can be slower; governed by diffusion + exchange reactions
Activation conditions [30]	Triggered automatically by crack (capsule rupture)	Often requires stimulus (thermal/light/electrical/solvent)
Healing efficiency trend [39]	High initial recovery; decreases as agent depletes	Moderate–high; can remain stable over cycles (if fatigue stable)
Dominant failure modes [40]	Agent depletion, incomplete filling, weak healed interface, capsule fatigue	Mobility suppression (high crosslink density/interphase confinement), bond exhaustion, aging
Scalability/manufacturing [41]	Capsule synthesis + dispersion; vascular complexity	Compatible with standard polymer processing; chemistry-specific
Mechanical shortcoming [42]	Capsules can reduce strength (defects); vascular voids	Network design can retain strength; fillers can reinforce
Environmental durability [43]	Aging of healing agent; leakage; compatibility issues	Bond exchange sensitivity to humidity/UV/oxidation
Nanofiller role [44]	Improves mechanical integrity; may hinder agent flow at high loading	Enables localized activation (Joule/photothermal), crack bridging
Best-fit applications [45]	Coatings, adhesives, low-load systems (single damage events)	Structural polymers, flexible electronics, fatigue-loaded components

**Table 3:** Influence of nanofiller type on reinforcement and healing behavior in self-healing polymer nanocomposites.

Nanofiller Type	Polymer Matrix	Primary Reinforcement Role	Effect on Healing	Functionality
Graphene/rGO [61]	Epoxy, PU, elastomers	High modulus, crack deflection	Improves healing efficiency, enables photothermal healing	Electrical & thermal conductivity
Carbon nanotubes [62]	Elastomers, hydrogels	Load transfer, percolation network	Enables electrical healing	Sensing, EMI shielding
Nano-clays [63]	Epoxy, coatings	Barrier and stiffness enhancement	Supports hydrogen-bond-based healing	Improved barrier properties
Silica nanoparticles [64]	Thermosets, coatings	Toughening, wear resistance	Stabilizes healing interfaces	Transparency, abrasion resistance
Metal oxides (Fe_3_O_4_, TiO_2_) [65]	Smart polymers	Thermal/magnetic response	Enables magnetic or photothermal healing	UV protection, catalytic effects

**Table 4:** Structure–property–healing design parameters controlling reinforcement–healability balance in self-healing polymer nanocomposites.

Parameter	Design Variable	Effect on Mechanical Properties	Effect on Healing Efficiency	Limitation
Crosslinking density [7]	Low to moderate	Moderate stiffness and strength	High healing efficiency (faster interdiffusion/bond exchange)	Reduced modulus at low crosslink density; poor load-bearing at elevated temperature
Nanofiller aspect ratio [81]	High aspect ratio fillers (CNTs, graphene)	High reinforcement and toughening at low filler loading	Improved crack closure and stimulus transduction (e.g., Joule/photothermal)	Agglomeration and dispersion difficulty; interphase immobilization at high loading
Filler loading [82]	Near percolation threshold	Increased modulus and multifunctionality	Maximum healing rate when stimulus delivery is optimized	Above percolation: mobility restriction and reduced repeatability due to confinement
Interfacial bonding [37]	Dynamic/reversible interface	Efficient stress transfer with reduced interfacial failure	Enhanced multi-cycle healing and durability	Requires tailored surface chemistry; synthesis complexity
Filler dispersion [83]	Uniform and stable dispersion	Homogeneous stress distribution; delayed crack propagation	Consistent healing performance across specimen	Processing challenges; viscosity increase and poor scalability at high filler fraction

**Table 5:** Quantitative trends in self-healing polymer nanocomposites: typical nanofiller loading windows, aspect ratio and percolation thresholds with performance outcomes.

Nanofiller Class	Typical Aspect Ratio (AR)	Typical Loading Window	Typical Percolation Threshold (Electrical)	Outcome on Mechanical Properties	Outcome on Healing Performance	Key Quantification
CNTs (MWCNT/SWCNT) [92]	10^2^–10^4^	~0.1–3 wt.%	~0.1–1 wt.%	Significant modulus/toughness gains at low φ	Fast Joule-assisted healing near φc; healing decreases at high φ	Use high AR CNTs to reach φc at low loading; avoid dense networks
Graphene/rGO [93]	10^2^–10^4^ (lateral)	~0.1–5 wt.%	~0.5–3 wt.%	Crack deflection; stiffness increase	Photothermal/Joule-assisted healing improves at moderate φ	Dispersion quality controls φc; avoid aggregation-induced confinement
MXene (2D) [94]	10^2^–10^3^	~0.5–10 wt.%	~1–5 wt.%	Strong stiffness + barrier enhancement	Excellent photothermal activation; mobility reduction at high φ	Maintain moderate φ to prevent over-densification of filler networks
Metallic nanowires (Ag/Cu) [95]	10^3^–10^5^	~0.2–5 wt.%	~0.1–1 wt.%	Conductive multifunctionality; reinforcement depends on interface	Very rapid Joule-triggered healing	Control hotspots; optimize φ slightly above φc
Silica/alumina nanoparticles (0D) [19]	~1–10	~1–15 wt.%	Not conductive	Increased modulus; toughening depends on adhesion	Healing may decrease if interphase becomes rigid	Use dynamic surface chemistry to preserve interphase mobility
Nanoclay/halloysite [96]	10^2^–10^3^	~1–10 wt.%	Not conductive	Modulus + barrier improvement	Healing depends on dispersion uniformity	Avoid high φ; confinement at high loading slows diffusion-driven healing

**Table 6:** Recommended minimum reporting checklist for standardized evaluation of self-healing polymer nanocomposites.

Category	Minimum Parameters
Polymer system [136]	Polymer type (thermoset/thermoplastic/elastomer/hydrogel), resin grade, curing agent type and ratio, mixing method, curing schedule (temperature–time), post-curing conditions
Dynamic healing chemistry (if intrinsic) [137]	Dynamic bond type (H-bond/disulfide/imine/Diels–Alder/vitrimer, etc.), catalyst (if any), reversible bond density (qualitative/quantitative), activation temperature or trigger condition
Extrinsic healing system (if used) [138]	Capsule/vascular type, shell chemistry, healing agent identity, catalyst identity/location, capsule size distribution, capsule loading, rupture mechanism and healing agent release conditions
Nanofiller identity [139]	Filler type (CNT/graphene/MXene/clay/SiO_2_, etc.), supplier/source, purity, particle size distribution, aspect ratio (for 1D/2D), surface area (if available), functionalization type
Nanofiller loading and composition [140]	Filler loading (wt% and/or vol%), hybrid filler ratios (if multiple), final composite composition table, density assumptions used for vol% conversion
Dispersion and microstructure [141]	Dispersion method (sonication/shear/three-roll milling), mixing energy/time, evidence of dispersion (SEM/TEM/AFM/Raman/XRD), agglomeration presence, orientation/alignment (if relevant)
Interfacial interactions/interphase [142]	Interface strategy (physical adsorption/covalent grafting/dynamic interface), coupling agent used, qualitative evidence (FTIR/XPS/Raman shift), interphase effect indicators (DMA/Tg shift)
Damage model and geometry [143]	Damage type (cut/scratch/notch/fatigue crack/delamination), sample geometry & thickness, notch length/width, crack length measurement method, number of damage cycles
Healing protocol [144]	Healing trigger (thermal/NIR/electrical/magnetic/solvent), healing temperature, time, pressure/contact method, environment (air/vacuum/humidity/water), number of healing cycles tested
Mechanical property reporting [145]	Property type (tensile/compressive/fracture toughness/peel/shear), strain rate, standard used (ASTM/ISO), ≥3 specimens minimum, error bars (SD), baseline vs. healed comparison
Healing efficiency metric [146]	Definition used (e.g., η = recovered strength/original strength × 100%), specify whether based on strength, toughness, modulus, fatigue life; report both initial and multicycle recovery
Stimuli-induced heating data (if trigger-based) [147]	Conductivity (electrical/thermal), trigger power/intensity, surface/bulk temperature profile during healing, time–temperature curve, efficiency of heating/photothermal conversion
Environmental durability [7]	Aging conditions (UV/humidity/thermal cycling/immersion), retention of healing after aging, filler leaching or stability (if relevant), failure mode changes after aging
Reproducibility & statistics [148]	Replicate count, statistical test (if used), uncertainty reporting, failure mode documentation, raw data availability (optional but recommended)

**Table 7:** Stimuli-responsive healing strategies in polymer nanocomposites and their activation mechanisms.

Stimulus Type	Nanofiller Role	Activation Mechanism	Healing Control	Applications
Thermal [169]	Heat conduction	Accelerates bond exchange	Moderate	Structural polymers
Light (IR/Vis) [170]	Photothermal conversion	Localized heating	High	Coatings, electronics
Electrical [171]	Joule heating	Resistive heat generation	Very high	Wearables, sensors
Magnetic [172]	Inductive heating	Alternating magnetic field	Wireless control	Embedded systems
Environmental (pH, moisture) [173]	Interface modulation	Reversible interactions	Passive	Coatings, membranes

**Table 8:** Application readiness assessment of self-healing polymer nanocomposites based on TRL and quantified performance gaps.

Application Area	TRL	Target Benchmark (Deployment Requirement)	Current Best Reported (Typical Literature Range)	Gap (%)/Limitation	Key Limiting Trade-Off
Protective coatings (anti-scratch, anti-corrosion) [179]	High	≥80% scratch closure within ≤10–60 min; strong adhesion after healing; weathering stability	Localized healing achievable (often ≥80% closure), but durability under abrasion/UV aging inconsistent	~10–30% gap in durability + weathering	Healing vs. hardness/abrasion resistance; filler aggregation under cycling
Adhesives/sealants [180]	High	≥70–90% joint strength recovery; repeatable healing (≥5 cycles); moisture stability	High recovery under thermal activation; multi-cycle retention often declines	~20–40% gap in multi-cycle retention	Crosslink density/strength vs. chain diffusion mobility
Flexible electronics/wearable conductors [181]	Medium–High	Conductivity recovery ≥90%; stable performance under bending (≥10^3^ cycles); low activation energy	Conductive healing demonstrated; property drift under cyclic strain	~10–25% gap in fatigue durability	Conductivity/percolation vs. stretchability and healing
Strain/pressure sensors (self-healing sensing layers) [182]	Medium	Signal recovery ≥ 90%; stable sensitivity after repeated damage; low-noise output	Good short-term recovery shown; baseline drift and hysteresis persist	~15–30% gap in signal stability	Filler network stability vs. self-healing rearrangement
Structural composites (transport/automotive) [53]	Medium	Retention of stiffness/strength ≥ 90%; fatigue crack growth suppression; repair without disassembly	Strength recovery often moderate; fatigue healing rarely quantified	~30–50% gap in fatigue performance	Reinforcement stiffness vs. healing mobility; interphase embrittlement
EMI shielding materials [183]	Medium	Shielding effectiveness ≥30–60 dB; conductivity retention after damage; multi-cycle healing	High EMI reported; mechanical reliability under repeated healing variable	~15–30% gap in mechanical durability	Conductive filler content vs. toughness/healing
Energy storage components (battery binders, solid polymer electrolytes) [184]	Emerging	Stable ionic conductivity; crack suppression; chemical/electrochemical stability	Healing concepts proposed; long-term cycling stability limited	~40–60% gap in long-term stability	Filler-ion transport coupling vs. mechanical integrity
Aerospace/defense composites [185]	Emerging	Certification-grade reliability; fatigue crack arrest; long-term environmental stability	Mostly lab-scale proof-of-concept	>50% gap (major)	Toughness + stiffness vs. repeatable healing; standardization/certification barrier
Biomedical devices (hydrogels, implants, patches) [173]	Emerging	Biocompatibility; low-temperature healing; sterilization stability	Strong potential; regulatory and toxicity concerns remain	~30–60% gap (regulatory + durability)	Healing chemistry vs. cytotoxicity; nanofiller fate

**Table 9:** Risk–mitigation overview for nanofillers used in self-healing polymer nanocomposites.

Nanofiller Type	Aging/Degradation Risk	Toxicity/Environmental Risk	Cost Level	Suggested Mitigation Strategy
CNTs [216]	Stress concentration and microcrack initiation under fatigue; network rearrangement during cycling	Potential inhalation hazard; persistence in environment if released	High	Encapsulation in polymer-rich interphase; surface functionalization; barrier coatings; wear-release testing
Graphene/rGO [217]	UV/thermal aging sensitivity depending on functional groups; interfacial debonding under load	Possible ecotoxicity concerns upon release; dust exposure	Medium–High	Covalent/noncovalent interfacial anchoring; platelet encapsulation; aging + abrasion release evaluation
MXenes [218]	Oxidation-sensitive; property drift under humidity/aging	Limited long-term toxicology data; environmental fate uncertain	High	Surface protection (antioxidant/polymer grafting); encapsulation; controlled humidity service design
Metal oxides (TiO_2_, ZnO, Fe_3_O_4_, Al_2_O_3_) [219]	Possible catalytic oxidation/photodegradation; embrittlement at high loading	Nanoparticle release risk under abrasion; regulatory scrutiny	Low–Medium	Use coated/functionalized oxides; optimize loading; UV stabilizers; accelerated aging protocols
Silica/clay nanofillers [220]	Generally stable; can increase brittleness if poorly dispersed	Lower toxicity risk; dust exposure possible	Low	Improve dispersion via compatibilizers; reduce agglomeration; optimize filler size and loading
Hybrid conductive networks (CNT + graphene, etc.) [221]	Enhanced percolation but increased interphase rigidity; fatigue sensitivity	Combined release risk; complex recycling	High	Gradient architectures (conductive layer localized); reversible interphase design; lifecycle assessment

## Data Availability

No new data were created or analyzed in this study. Data sharing is not applicable to this article.

## References

[1] Desai S.K., Bera S., Mondal D. (2019). Multifaceted synthesis, properties and applications of polyurethanes and its composites. Curr. Org. Chem..

[2] Agrawal N., Arora B. (2022). Self-healing polymers and composites: Extrinsic routes. Mini-Rev. Org. Chem..

[3] Zhou H., Li C., Zhang Y., Tu W., Li Q., Peng Y., Zhang Z., Zheng Z. (2025). Nano-SiO_2_/PVC Composite Material: A Review on Modification, Preparation, Properties, and Applications. J. Appl. Polym. Sci..

[4] Cheng X., Huang W., Zhang Q. (2025). Damage and Failure Analysis of Polymer-Based Composites. Polymers.

[5] Naebe M., Abolhasani M.M., Khayyam H., Amini A., Fox B. (2016). Crack damage in polymers and composites: A review. Polym. Rev..

[6] Naga S.A., El-Sayed T.A. (2024). Fatigue failure in polymeric materials: Insights from experimental testing. J. Fail. Anal. Prev..

[7] Ekeocha J., Ellingford C., Pan M., Wemyss A.M., Bowen C., Wan C. (2021). Challenges and opportunities of self-healing polymers and devices for extreme and hostile environments. Adv. Mater..

[8] Srivastav R.S., More A.P. (2025). A Comprehensive Review of Self-Healing Polymers: Mechanisms, Types, and Industry Implications. Polym. Adv. Technol..

[9] Priyadarsini M., Sahoo D.R., Biswal T. (2021). A new generation self-healing composite materials. Mater. Today Proc..

[10] Kontiza A., Kartsonakis I.A. (2024). Smart composite materials with self-healing properties: A review on design and applications. Polymers.

[11] Ratwani C.R., Novoselov K.S., Abdelkader A.M. (2025). Applications and mechanistic insights into intrinsically self-healing polymers with multifunctional 2D materials. SusMat.

[12] Liu Z., Zhong Y., Li S., Yu S., Zhong J., Yang Y., Shen L. (2024). Hybrid Self-Repairing Polymer Composites Based on a Mixture of Intrinsic and Extrinsic Self-Healing. Macromol. Chem. Phys..

[13] Kessler M.R., Sottos N.R., White S.R. (2003). Self-healing structural composite materials. Compos. Part A Appl. Sci. Manuf..

[14] Tazwar M.F., Muhtasim S.T., Reza T., Autul Y.S., Hoque M.E. (2023). Inorganic Nanofiller-Incorporated Polymeric Nanocomposites for Biomedical Applications. Nanofillers.

[15] Kausar A. (2020). A review of fundamental principles and applications of polymer nanocomposites filled with both nanoclay and nano-sized carbon allotropes–graphene and carbon nanotubes. J. Plast. Film Sheeting.

[16] Das P.P., Chaudhary V., Ahmad F., Manral A. (2021). Effect of nanotoxicity and enhancement in performance of polymer composites using nanofillers: A state-of-the-art review. Polym. Compos..

[17] Hornat C.C., Urban M.W. (2020). Shape memory effects in self-healing polymers. Prog. Polym. Sci..

[18] Kanu N.J., Gupta E., Vates U.K., Singh G.K. (2019). Self-healing composites: A state-of-the-art review. Compos. Part A Appl. Sci. Manuf..

[19] Orellana J., Moreno-Villoslada I., Bose R.K., Picchioni F., Flores M.E., Araya-Hermosilla R. (2021). Self-healing polymer nanocomposite materials by Joule effect. Polymers.

[20] Zhou S., Qi N., Zhang Z., Jiang P., Li A., Lu Y., Su X. (2025). Recent progress in intrinsic self-healing polymer materials: Mechanisms, challenges and potential applications in oil and gas development. Chem. Eng. J..

[21] Schenk V., Labastie K., Destarac M., Olivier P., Guerre M. (2022). Vitrimer composites: Current status and future challenges. Mater. Adv..

[22] Van Zee N.J., Nicolaÿ R. (2020). Vitrimers: Permanently crosslinked polymers with dynamic network topology. Prog. Polym. Sci..

[23] Irzhak V.I., Uflyand I.E., Dzhardimalieva G.I. (2022). Self-healing of polymers and polymer composites. Polymers.

[24] Peng Y., Gu S., Wu Q., Xie Z., Wu J. (2023). High-performance self-healing polymers. Acc. Mater. Res..

[25] Boruah J.S., Chowdhury D. (2022). Advances in carbon nanomaterial–clay nanocomposites for diverse applications. Minerals.

[26] Golonka M., Laska J. (2025). Microcapsules in self-healing materials: A review. Smart Mater. Struct..

[27] Patrick J.F., Robb M.J., Sottos N.R., Moore J.S., White S.R. (2016). Polymers with autonomous life-cycle control. Nature.

[28] Patrick J.F., Hart K.R., Krull B.P., Diesendruck C.E., Moore J.S., White S.R., Sottos N.R. (2014). Continuous self-healing life cycle in vascularized structural composites. Adv. Mater..

[29] Şahin E., Boztoprak Y., Yazıcı M. (2025). Development of Self-Healing Thermoplastic Composites With Reactive Thermoplastic Agent-Filled Macrocapsules. J. Appl. Polym. Sci..

[30] McDonald S.A., Coban S.B., Sottos N.R., Withers P.J. (2019). Tracking capsule activation and crack healing in a microcapsule-based self-healing polymer. Sci. Rep..

[31] Bah M.G., Bilal H.M., Wang J. (2020). Fabrication and application of complex microcapsules: A review. Soft Matter.

[32] Li B., Cao P.F., Saito T., Sokolov A.P. (2022). Intrinsically self-healing polymers: From mechanistic insight to current challenges. Chem. Rev..

[33] Hia I.L., Vahedi V., Pasbakhsh P. (2016). Self-healing polymer composites: Prospects, challenges, and applications. Polym. Rev..

[34] Khan S.W., Tariq M. (2022). Recent Advances in Self-Healing Green Composites for Structural Applications. New Environ. Friendly Mater..

[35] Bhuyan M.M., Jeong J.H. (2025). Preparation of Hydrogel by Crosslinking and Multi-Dimensional Applications. Gels.

[36] Feiteira J., Gruyaert E., De Belie N. (2016). Self-healing of moving cracks in concrete by means of encapsulated polymer precursors. Constr. Build. Mater..

[37] Paul S., Kaushik A.K., Jain A., Banerjee S. (2025). Intelligent self-healing polymeric systems for functional and durable coatings. Chem. Asian J..

[38] Achilias D.S. (2007). A review of modeling of diffusion controlled polymerization reactions. Macromol. Theory Simul..

[39] Liu T., Li C., Yao H., Sun F., Wang L., Yao B., Xu J., Fu J. (2023). Extremely strengthening fatigue resistance, elastic restorability and thermodynamic stability of a soft transparent self-healing network based on a dynamic molecular confinement-induced bioinspired nanostructure. Mater. Horiz..

[40] Wu X.F., Yarin A.L. (2013). Recent progress in interfacial toughening and damage self-healing of polymer composites based on electrospun and solution-blown nanofibers: An overview. J. Appl. Polym. Sci..

[41] Hansoge N.K., Gupta A., White H., Giuntoli A., Keten S. (2021). Universal relation for effective interaction between polymer-grafted nanoparticles. Macromolecules.

[42] Shields Y., De Belie N., Jefferson A., Van Tittelboom K. (2021). A review of vascular networks for self-healing applications. Smart Mater. Struct..

[43] Li F., Xu Z., Hu H., Kong Z., Chen C., Tian Y., Zhang W., Ying W.B., Zhang R., Zhu J. (2021). A polyurethane integrating self-healing, anti-aging and controlled degradation for durable and eco-friendly E-skin. Chem. Eng. J..

[44] Guchait A., Saxena A., Chattopadhyay S., Mondal T. (2022). Influence of nanofillers on adhesion properties of polymeric composites. ACS Omega.

[45] Pandey S.K., Mishra S., Ghosh S., Rohan R., Maji P.K. (2024). Self-healing polymers for aviation applications and their impact on circular economy. Polym. Eng. Sci..

[46] Liu Y., Chang X., Liu S., Qu Y., Yu W., Wang W., Liu W., Sun S., Yu Q. (2025). Modified Zirconia Nanoparticle-Glass Fiber Reinforced PEEK for Pole Frames in Alkaline Water Electrolysers. J. Appl. Polym. Sci..

[47] Idumah C.I., Hassan A., Ogbu J., Ndem J.U., Nwuzor I.C. (2019). Recently emerging advancements in halloysite nanotubes polymer nanocomposites. Compos. Interfaces.

[48] Chakma P., Konkolewicz D. (2019). Dynamic covalent bonds in polymeric materials. Angew. Chem. Int. Ed..

[49] Ha Y.M., Kim Y.N., Jung Y.C. (2021). Rapid and local self-healing ability of polyurethane nanocomposites using photothermal polydopamine-coated graphene oxide triggered by near-infrared laser. Polymers.

[50] Zhang M.Q., Rong M.Z. (2022). Extrinsic and Intrinsic Approaches to Self-Healing Polymers and Polymer Composites.

[51] Bond I.P., Trask R.S., Williams H.R. (2008). Self-healing fiber-reinforced polymer composites. MRS Bull..

[52] Tan Y.J., Susanto G.J., Anwar Ali H.P., Tee B.C. (2021). Progress and roadmap for intelligent self-healing materials in autonomous robotics. Adv. Mater..

[53] Khan A., Jawaid M., Raveendran S.N., Asiri A.M.A. (2019). Self-Healing Composite Materials: From Design to Applications.

[54] Caruso M.M., Davis D.A., Shen Q., Odom S.A., Sottos N.R., White S.R., Moore J.S. (2009). Mechanically-induced chemical changes in polymeric materials. Chem. Rev..

[55] Mohamadhoseini M., Mohamadnia Z. (2025). Advances in β-Cyclodextrin-Driven Self-Healing Materials: Molecular Design and Multifunctional Applications. Adv. Mater..

[56] Zhao F., Yuan W., Chen H., Fu H., Li Z., Xiao J., Feng Y. (2025). Advances in Organic Porous Polymeric-Supported Photothermal Phase Change Materials. Carbon Energy.

[57] Lubineau G., Alfano M., Tao R., Wagih A., Yudhanto A., Li X., Almuhammadi K., Hashem M., Hu P., Mahmoud H.A. (2024). Harnessing Extrinsic Dissipation to Enhance the Toughness of Composites and Composite Joints: A State-of-the-Art Review of Recent Advances. Adv. Mater..

[58] Song W., Wen Y., Cho Y., Zhang X., Kang D., Shin E., Yu D.G., Li G., Liao Y., Kim I.D. (2025). Advances and Prospects in Multifunctional Composite Fibrous Materials Utilizing Porous Organic Polymers. Adv. Mater..

[59] Sabet M. (2024). Unveiling advanced self-healing mechanisms in graphene polymer composites for next-generation applications in aerospace, automotive, and electronics. Polym. Plast. Technol. Mater..

[60] Qamar I.P., Sottos N.R., Trask R.S. (2020). Grand challenges in the design and manufacture of vascular self-healing. Multifunct. Mater..

[61] Roh S., Nam Y., Nguyen M.T.N., Han J.H., Lee J.S. (2024). Dynamic covalent bond-based polymer chains operating reversibly with temperature changes. Molecules.

[62] Men G., Niu W., Liu X. (2025). Hydrogen-Bond-Mediated Polymers: Strengthening, Toughening, and Stabilizing Effects. Chem. –A Eur. J..

[63] Islam S., Bhat G. (2021). Progress and challenges in self-healing composite materials. Mater. Adv..

[64] Shen Y., Jin J., Su Y., Chen M., Wu L. (2025). Developments and Opportunities in Temperature-Responsive Thermal Smart Materials. Adv. Mater..

[65] Vahedi V., Pasbakhsh P. (2015). Polymer nanocomposites reinforced by halloysite nanotubes: A review. Natural Mineral Nanotubes: Properties and Applications.

[66] Mihashi H., Nishiwaki T. (2012). Development of engineered self-healing and self-repairing concrete-state-of-the-art report. J. Adv. Concr. Technol..

[67] Zhi C., Song J., Chen J., Liu H., Hu J., Tian Z., Wong W.Y., Yan F. (2025). Emerging Gel-Based Organic Electrochemical Transistors: Device Design, Engineering, and Applications. Adv. Funct. Mater..

[68] Chan J.X., Wong J.F., Petrů M., Hassan A., Nirmal U., Othman N., Ilyas R.A. (2021). Effect of nanofillers on tribological properties of polymer nanocomposites: A review on recent development. Polymers.

[69] Bakis G., Kothmann M.H., Zeiler R., Brückner A., Ziadeh M., Breu J., Altstädt V. (2018). Influence of size, aspect ratio and shear stiffness of nanoclays on the fatigue crack propagation behavior of their epoxy nanocomposites. Polymer.

[70] Roy D., Mandal S., Chandrakar K., Dwivedi M. (2025). Controlling Porosity and Multifunctionality in Electrospun Polymeric Fibers by Nanoscale Phase Separations: Flory–Huggins Interaction Parameters Revisited. Macromol. Chem. Phys..

[71] Liu Y., Chen L., Li W., Pu J., Wang Z., He B., Yuan S., Xin J., Huang L., Luo Z. (2024). Scalable production of functional fibers with nanoscale features for smart textiles. ACS Nano.

[72] Ma Z., Wang W., Xiong Y., Long Y., Shao Q., Wu L., Wang J., Tian P., Khan A.U., Yang W. (2025). Carbon micro/nano machining toward miniaturized device: Structural engineering, large-scale fabrication, and performance optimization. Small.

[73] Bhattacharya S., Samanta S.K. (2016). Soft-nanocomposites of nanoparticles and nanocarbons with supramolecular and polymer gels and their applications. Chem. Rev..

[74] Kim G., Caglayan C., Yun G.J. (2024). High Modulus Epoxy/GO-PANI Self-Healing Materials Without Catalyst by Molecular Engineering and Nanocomposite Fabrication. Polymers.

[75] Wang T., Wang W., Feng H., Sun T., Ma C., Cao L., Qin X., Lei Y., Piao J., Feng C. (2022). Photothermal nanofiller-based polydimethylsiloxane anticorrosion coating with multiple cyclic self-healing and long-term self-healing performance. Chem. Eng. J..

[76] Hemath M., Mavinkere Rangappa S., Kushvaha V., Dhakal H.N., Siengchin S. (2020). A comprehensive review on mechanical, electromagnetic radiation shielding, and thermal conductivity of fibers/inorganic fillers reinforced hybrid polymer composites. Polym. Compos..

[77] Li X., Xu W., Xin Y., Yuan J., Ji Y., Chu S., Liu J., Luo Q. (2021). Supramolecular Polymer Nanocomposites for Biomedical Applications. Polymers.

[78] Mittal V. (2009). Polymer layered silicate nanocomposites: A review. Materials.

[79] Lin Q., Kundu D., Skyllas-Kazacos M., Lu J., Zhao D., Amine K., Dai L., Wang D.W. (2024). Perspective on Lewis Acid-Base Interactions in Emerging Batteries. Adv. Mater..

[80] Kartsonakis I.A., Kontiza A., Kanellopoulou I.A. (2024). Advanced micro/nanocapsules for self-healing coatings. Appl. Sci..

[81] Chen J., Gao X., Song W. (2019). Effect of various carbon nanofillers and different filler aspect ratios on the thermal conductivity of epoxy matrix nanocomposites. Results Phys..

[82] Mochane M.J., Sefadi J.S., Motsoeneng T.S., Mokoena T.E., Mofokeng T.G., Mokhena T.C. (2020). The effect of filler localization on the properties of biopolymer blends, recent advances: A review. Polym. Compos..

[83] Rahman M.M., Khan K.H., Parvez M.M.H., Irizarry N., Uddin M.N. (2025). Polymer nanocomposites with optimized nanoparticle dispersion and enhanced functionalities for industrial applications. Processes.

[84] Kim K.H., Ong J.L., Okuno O. (2002). The effect of filler loading and morphology on the mechanical properties of contemporary composites. J. Prosthet. Dent..

[85] Chen J., Zhao N., Li M., Wang Y., Gao W., Bai H. (2025). Horn-Inspired Hierarchical Tubular Composites for Recoverable High-Energy Absorption. Adv. Mater..

[86] Zhang Z., Hu L., Wang R., Zhang S., Fu L., Li M., Xiao Q. (2024). Advances in Monte Carlo method for simulating the electrical percolation behavior of conductive polymer composites with a carbon-based filling. Polymers.

[87] Haghgoo M., Ansari R., Hassanzadeh-Aghdam M.K. (2021). Synergic effect of graphene nanoplatelets and carbon nanotubes on the electrical resistivity and percolation threshold of polymer hybrid nanocomposites. Eur. Phys. J. Plus.

[88] Xu D., Li Z., Li L., Wang J. (2020). Insights into the photothermal conversion of 2D MXene nanomaterials: Synthesis, mechanism, and applications. Adv. Funct. Mater..

[89] Champagne J., Pang S.S., Li G. (2016). Effect of confinement level and local heating on healing efficiency of self-healing particulate composites. Compos. Part B Eng..

[90] Li J., Ma P.C., Chow W.S., To C.K., Tang B.Z., Kim J.K. (2007). Correlations between percolation threshold, dispersion state, and aspect ratio of carbon nanotubes. Adv. Funct. Mater..

[91] Shah P.K., Stansbury J.W. (2014). Role of filler and functional group conversion in the evolution of properties in polymeric dental restoratives. Dent. Mater..

[92] Bauhofer W., Kovacs J.Z. (2009). A review and analysis of electrical percolation in carbon nanotube polymer composites. Compos. Sci. Technol..

[93] Lalire T., Longuet C., Taguet A. (2024). Electrical properties of graphene/multiphase polymer nanocomposites: A review. Carbon.

[94] FitzPatrick J., Gogotsi Y. (2025). MXene polymer composites: A review. MRS Bull..

[95] Bril’ I.Y., Voronin A., Fadeev Y., Pavlikov A., Govorun I., Podshivalov I., Parshin B., Makeev M., Mikhalev P., Afanasova K. (2024). Laser-Induced Silver Nanowires/Polymer Composites for Flexible Electronics and Electromagnetic Compatibility Application. Polymers.

[96] Ibrahim A., Klopocinska A., Horvat K., Abdel Hamid Z. (2021). Graphene-based nanocomposites: Synthesis, mechanical properties, and characterizations. Polymers.

[97] Wang Y., Zhou Z., Li S., Zheng H., Lu J., Wang S., Zhang J., Wang K., Lin K. (2022). Near-infrared-light-assisted self-healing graphene-thermopolyurethane composite films. Polymers.

[98] Díez-García I., Eceiza A., Tercjak A. (2019). Improvement of mechanical properties and self-healing efficiency by ex-situ incorporation of TiO2 nanoparticles to a waterborne poly (urethane-urea). Polymers.

[99] Wang Y., Zhou Z., Chen J., Li S., Zheng H., Lu J., Wang S., Zhang J., Lin K., Wang K. (2022). Self-Healing Silver Nanowires and Reduced Graphene Oxide/Polyurethane Composite Film Based on the Diels–Alder Reaction under Infrared Radiation. Membranes.

[100] Jamil H., Faizan M., Adeel M., Jesionowski T., Boczkaj G., Balčiūnaitė A. (2024). Recent advances in polymer nanocomposites: Unveiling the frontier of shape memory and self-healing properties—A comprehensive review. Molecules.

[101] Zheng X., Nie W., Guo Y., Douglas J.F., Xia W. (2023). Influence of chain stiffness on the segmental dynamics and mechanical properties of cross-linked polymers. Macromolecules.

[102] Gopalakrishnan K., Mishra P. (2024). Self-healing polymer a dynamic solution in food industry: A comprehensive review. Food Biophys..

[103] Deng J., Jin Y., Li Z., Xue M., Zhang Y., Dong A., Burenjargal M. (2025). self-healing materials and their applications. J. Polym. Res..

[104] Kim S., Shin Y., Han J., Kim H.J., Sunwoo S.H. (2024). Introductory review of soft implantable bioelectronics using conductive and functional hydrogels and hydrogel nanocomposites. Gels.

[105] Valenzuela C., Chen Y., Wang L., Feng W. (2022). Functional liquid crystal elastomers based on dynamic covalent chemistry. Chem. A Eur. J..

[106] Zhao W., Su Y., Wang D. (2017). Synergetic effects of interfacial and spatial confinement in polymer nanocomposites. Mod. Phys. Lett. B.

[107] Kacmaz N., Macit C.K., Gurgenc E., Biryan F., Ilkilic C., Gurgenc T. (2025). Graphite-doped cadmium oxide nanoparticles: Structural modifications, dielectric enhancement, and percolation-driven electrical evolution. J. Mater. Sci. Mater. Electron..

[108] Sang Z., Zhou Q., Rajagopalan K.K., Thomas E.L., Gardea F., Sukhishvili S.A. (2022). Dynamic polymer network conductive Nanocomposites: Low percolation threshold and Joule-heating-induced network plasticity. Chem. Eng. J..

[109] Sangroniz L., Landa M., Fernández M., Santamaria A. (2021). Matching rheology, conductivity and joule effect in PU/CNT nanocomposites. Polymers.

[110] Guadagno L., Vertuccio L., Naddeo C., Calabrese E., Barra G., Raimondo M., Sorrentino A., Binder W.H., Michael P., Rana S. (2019). Reversible self-healing carbon-based nanocomposites for structural applications. Polymers.

[111] Qin X., Tian Y., Zhao H., Wang J., Zhan S., Qu J., Zhang L., Liu J. (2025). Toward Reprocessable High-Performance Elastomer: Self-Assembly, Dynamic Covalent Chemistry, and Tailorable Properties. Adv. Mater..

[112] Lei H., Fan D. (2021). Conductive, adaptive, multifunctional hydrogel combined with electrical stimulation for deep wound repair. Chem. Eng. J..

[113] Xu C., Chen Y., Zhao S., Li D., Tang X., Zhang H., Huang J., Guo Z., Liu W. (2024). Mechanical regulation of polymer gels. Chem. Rev..

[114] Xiao J., Chen Y., Cheng L., Wu H., Hou M., Ma L., Chen X., Wong C.P. (2025). Flash Joule Heating: A Transformative Non-Equilibrium Strategy for Next-Generation Advanced Materials. Small Methods.

[115] Siddique J.A., Ahmad A., Mohd A. (2018). Self healing materials and conductivity. Electrically Conductive Polymer and Polymer Composites: From Synthesis to Biomedical Applications.

[116] Zheng N., Xu Y., Zhao Q., Xie T. (2021). Dynamic covalent polymer networks: A molecular platform for designing functions beyond chemical recycling and self-healing. Chem. Rev..

[117] Šupová M., Martynková G.S., Barabaszová K. (2011). Effect of nanofillers dispersion in polymer matrices: A review. Sci. Adv. Mater..

[118] Li Q., Cheng S. (2020). Polymer nanocomposites for high-energy-density capacitor dielectrics: Fundamentals and recent progress. IEEE Electr. Insul. Mag..

[119] Cohades A., Hostettler N., Pauchard M., Plummer C.J., Michaud V. (2018). Stitched shape memory alloy wires enhance damage recovery in self-healing fibre-reinforced polymer composites. Compos. Sci. Technol..

[120] Fisher C.R., Henderson H.B., Kesler M.S., Zhu P., Bean G.E., Wright M.C., Newman J.A., Brinson L.C., Figueroa O., Manuel M.V. (2018). Repairing large cracks and reversing fatigue damage in structural metals. Appl. Mater. Today.

[121] Kuang W., Schwartz B., Mather P.T. (2025). Electrospun Polyurethane Blends Exhibiting Shape Memory and Self-Healing Properties. J. Polym. Sci..

[122] Voyiadjis G.Z., Kattan P.I. (2017). Mechanics of damage, healing, damageability, and integrity of materials: A conceptual framework. Int. J. Damage Mech..

[123] Mezzomo L., Ferrara C., Brugnetti G., Callegari D., Quartarone E., Mustarelli P., Ruffo R. (2020). Exploiting self-healing in lithium batteries: Strategies for next-generation energy storage devices. Adv. Energy Mater..

[124] Yahia A.K.M., Shahjalal M. (2025). Recent developments and challenges in fracture mechanics–based fatigue life prediction. ASRC Procedia Glob. Perspect. Sci. Scholarsh..

[125] Devi R., Gupta P., Khatua C., Naskar K., Chattopadhyay S. (2025). Thermoplastic Polyurethane-Based Stimuli-Responsive Nanocomposites: A Review on Self-Healing and Shape Memory Properties. Polym. Eng. Sci..

[126] Pandey A., Sharma A.K., Shukla D.K., Pandey K.N. (2023). Effect of self-healing by dicyclopentadiene microcapsules on tensile and fatigue properties of epoxy composites. Materials.

[127] Verma A., Bhushan K., Singh H. (2025). Nanocomposites for extrinsic self-healing polymer materials: A comprehensive review of their repair behaviour. Results Chem..

[128] Muhammad N.Z., Shafaghat A., Keyvanfar A., Majid M.Z.A., Ghoshal S.K., Yasouj S.E.M., Ganiyu A.A., Kouchaksaraei M.S., Kamyab H., Taheri M.M. (2016). Tests and methods of evaluating the self-healing efficiency of concrete: A review. Constr. Build. Mater..

[129] Okay O. (2020). How to design both mechanically strong and self-healable hydrogels?. Self-Healing and Self-Recovering Hydrogels.

[130] Jiang J.X., Jia X.Y., Zhu D.Y., Qiu X., Lan M.H., Li C., Chen S., Liu W., Chen L., Liu Q. (2025). Regulation of Lignin Microstructures to Construct Fully Biomass-Based Elastomers for Large Crack Self-Healing Artificial Muscles. Small.

[131] Hohlbein N., Shaaban A., Bras A.R., Pyckhout-Hintzen W., Schmidt A.M. (2015). Self-healing dynamic bond-based rubbers: Understanding the mechanisms in ionomeric elastomer model systems. Phys. Chem. Chem. Phys..

[132] Demiral M. (2025). Strength in adhesion: A multi-mechanics review covering tensile, shear, fracture, fatigue, creep, and impact behavior of polymer bonding in composites. Polymers.

[133] Wang S., Zhang Z., Li Z., Zhang Z., Islam M.Z., Jiang X., Gao H., Xu S., Dong Y. (2024). Ultra-fast light repair, ultrasensitive, large strain detection range PDA@ RGO/EVA composites fiber flexible strain sensor. Sens. Actuators A Phys..

[134] Gautam A., Sarkar P.K. (2019). Continuum damage mechanics-based ductile behavior and fatigue life estimation of low carbon steels: AISI 1020 and AISI 1030. Proc. Inst. Mech. Eng. Part L J. Mater. Des. Appl..

[135] Cordoba A., Gutiérrez-Mejía F.A., Cepeda-Granados G., Cauich-Rodríguez J.V., Esquivel Escalante K. (2025). Self-Healing Polymer-Based Coatings: Mechanisms and Applications Across Protective and Biofunctional Interfaces. Polymers.

[136] Yao Y., Wang J., Lu H., Xu B., Fu Y., Liu Y., Leng J. (2015). Thermosetting epoxy resin/thermoplastic system with combined shape memory and self-healing properties. Smart Mater. Struct..

[137] Roy N., Bruchmann B., Lehn J.M. (2015). DYNAMERS: Dynamic polymers as self-healing materials. Chem. Soc. Rev..

[138] Malekkhouyan R., Neisiany R.E., Khorasani S.N., Das O., Berto F., Ramakrishna S. (2021). The influence of size and healing content on the performance of extrinsic self-healing coatings. J. Appl. Polym. Sci..

[139] Bronnikov S., Kostromin S., Asandulesa M., Podshivalov A., Timpu D. (2019). Morphology, structure, and segmental dynamics in polyazomethine/hybrid carbon nanofillers composites. Polym. Compos..

[140] Singh D., Sotiriou G.A., Zhang F., Mead J., Bello D., Wohlleben W., Demokritou P. (2016). End-of-life thermal decomposition of nano-enabled polymers: Effect of nanofiller loading and polymer matrix on by-products. Environ. Sci. Nano.

[141] Łukowski P., Adamczewski G. (2013). Self-repairing of polymer-cement concrete. Bull. Pol. Acad. Sci. Tech. Sci..

[142] Zhang Y., Zhang Y., Deng J., Xue R., Yang S., Ma Y., Wang Z. (2024). In situ electrochemically-bonded self-adapting polymeric interface for durable aqueous zinc ion batteries. Adv. Funct. Mater..

[143] Zhang M.Q., Rong M.Z. (2012). Theoretical consideration and modeling of self-healing polymers. J. Polym. Sci. Part B Polym. Phys..

[144] Blaiszik B.J., Kramer S.L., Olugebefola S.C., Moore J.S., Sottos N.R., White S.R. (2010). Self-healing polymers and composites. Annu. Rev. Mater. Res..

[145] Son D.H., Kim G.Y., Jeong J.E., Lee S.H., Park Y.I., Kong H., Cheong I.W., Kim J.C. (2021). Influence of material properties on the damage-reporting and self-healing performance of a mechanically active dynamic network polymer in coating applications. Molecules.

[146] Tan P.S., Somashekar A.A., Casari P., Bhattacharyya D. (2019). Healing efficiency characterization of self-repairing polymer composites based on damage continuum mechanics. Compos. Struct..

[147] Zhang H., Zhao Y. (2013). Polymers with dual light-triggered functions of shape memory and healing using gold nanoparticles. ACS Appl. Mater. Interfaces.

[148] Abend M., Tianis L., Kunz C., Zechel S., Gräf S., Müller F.A., Schubert U.S., Hager M.D. (2020). A novel approach for the quantification of scratch healing of polymers. Polym. Test..

[149] Bisht N., Vishwakarma J., Jaiswal S., Kumar P., Srivastava A.K., Dhand C., Dwivedi N. (2025). Synergizing chemistry: Unveiling the potential of hybrid fillers for enhanced performance in shape memory polymers. Adv. Compos. Hybrid Mater..

[150] Jain A., Goswami S., Banerjee S. (2025). Advances in Self-Healing Polymers: Mechanisms, Applications, and Future Perspectives. Macromol. Chem. Phys..

[151] Yuan Q., Chen J., Shi C., Shi X., Sun C., Jiang B. (2025). Advances in Self-Healing Perovskite Solar Cells Enabled by Dynamic Polymer Bonds. Macromol. Rapid Commun..

[152] Zulkefli N.A., Mustapha R., Jusoh S.M., Ruzaidi Ghazali C.M., Awang M., Norrrahim M.N.F., Ilyas R.A. (2023). Hybrid nanofiller reinforcement in thermoset and biothermoset applications: A review. Nanotechnol. Rev..

[153] Wypych G. (2022). Self-Healing Materials: Principles and Technology.

[154] Zhou Y., Li L., Han Z., Li Q., He J., Wang Q. (2022). Self-healing polymers for electronics and energy devices. Chem. Rev..

[155] Mohd Sani N.F., Shuib R.K., Zainol M.H., Othman N., Tran D.L. (2024). Effect of multi-walled carbon nanotubes reinforcement on self-healing performance of natural rubber. Pure Appl. Chem..

[156] Bilotti E., Zhang H., Deng H., Zhang R., Fu Q., Peijs T. (2013). Controlling the dynamic percolation of carbon nanotube based conductive polymer composites by addition of secondary nanofillers: The effect on electrical conductivity and tuneable sensing behaviour. Compos. Sci. Technol..

[157] Iravani S., Zarepour A., Khosravi A., Varma R.S., Zarrabi A. (2025). Smart MXene-based microrobots for targeted drug delivery and synergistic therapies. Nanoscale.

[158] Cho S.H., Andersson H.M., White S.R., Sottos N.R., Braun P.V. (2006). Polydimethylsiloxane-based self-healing materials. Adv. Mater..

[159] Guo Y., Zou D., Zhu W., Yang X., Zhao P., Chen C., Shuai M. (2019). Infrared induced repeatable self-healing and removability of mechanically enhanced graphene–epoxy flexible materials. RSC Adv..

[160] Cerdan K., Moya C., Van Puyvelde P., Bruylants G., Brancart J. (2022). Magnetic self-healing composites: Synthesis and applications. Molecules.

[161] Wang M., Deng Z., Guo Y., Xu P. (2023). Engineering functional natural polymer-based nanocomposite hydrogels for wound healing. Nanoscale Adv..

[162] Wen N., Song T., Ji Z., Jiang D., Wu Z., Wang Y., Guo Z. (2021). Recent advancements in self-healing materials: Mechanicals, performances and features. React. Funct. Polym..

[163] Kim S., Jeon H., Koo J.M., Oh D.X., Park J. (2024). Practical applications of self-healing polymers beyond mechanical and electrical recovery. Adv. Sci..

[164] Guadagno L., Naddeo C., Raimondo M., Barra G., Vertuccio L., Sorrentino A., Binder W.H., Kadlec M. (2017). Development of self-healing multifunctional materials. Compos. Part B Eng..

[165] An D., Cheng S., Zhang Z., Jiang C., Fang H., Li J., Liu Y., Wong C.P. (2019). A polymer-based thermal management material with enhanced thermal conductivity by introducing three-dimensional networks and covalent bond connections. Carbon.

[166] Araujo M., Van Vlierberghe S., Feiteira J., Graulus G.J., Van Tittelboom K., Martins J.C., Dubruel P., De Belie N. (2016). Cross-linkable polyethers as healing/sealing agents for self-healing of cementitious materials. Mater. Des..

[167] Zhong N., Post W. (2015). Self-repair of structural and functional composites with intrinsically self-healing polymer matrices: A review. Compos. Part A Appl. Sci. Manuf..

[168] Ramesh M., Bhuvaneswari V., Balaji D., Rajeshkumar L. (2022). Self-healable conductive and polymeric composite materials. Aerosp. Polym. Mater..

[169] Wang Y., Pham D.T., Ji C. (2015). Self-healing composites: A review. Cogent Eng..

[170] Idumah C.I. (2021). Novel trends in self-healable polymer nanocomposites. J. Thermoplast. Compos. Mater..

[171] Altuna F.I., Hoppe C.E. (2021). Self-Healing Polymer Coatings. Self-Healing Smart Materials and Allied Applications.

[172] Li S., Zhou X., Dong Y., Li J. (2020). Flexible self-repairing materials for wearable sensing applications: Elastomers and hydrogels. Macromol. Rapid Commun..

[173] Idumah C.I. (2021). Recent advancements in self-healing polymers, polymer blends, and nanocomposites. Polym. Polym. Compos..

[174] Trask R.S., Williams H.R., Bond I.P. (2007). Self-healing polymer composites: Mimicking nature to enhance performance. Bioinspiration Biomim..

[175] Cioffi M.O.H., Bomfim A.S., Ambrogi V., Advani S.G. (2022). A review on self-healing polymers and polymer composites for structural applications. Polym. Compos..

[176] JE P.C., Sultan M.T., Selvan C.P., Irulappasamy S., Mustapha F., Basri A.A., Safri S.N. (2020). Manufacturing challenges in self-healing technology for polymer composites—A review. J. Mater. Res. Technol..

[177] Li Y., Chen S., Li X., Wu M., Sun J. (2015). Highly transparent, nanofiller-reinforced scratch-resistant polymeric composite films capable of healing scratches. ACS Nano.

[178] Dolui T., Natarajan T.S., S A., Chanda J., Ghosh P., Mukhopadhyay R., Wießner S., Heinrich G., Das A., Banerjee S.S. (2023). Stimuli–responsive Mechanoadaptive elastomeric composite materials: Challenges, opportunities, and new approaches. Adv. Eng. Mater..

[179] Beyrami H., Golshan M., Zardehi-Tabriz A., Salami-Kalajahi M. (2025). Smart coatings: Fundamentals, preparation approaches, and applications. Adv. Mater. Technol..

[180] Mauldin T.C., Kessler M.R. (2010). Self-healing polymers and composites. Int. Mater. Rev..

[181] Huang Z., Guo Z., Zhou H., Liu Q., Ma Q., Ge C., Wei S., Pan K., Alahi M.E.E., Zeng Q. (2025). Liquid Metal-Based Soft Materials for Self-Healing Flexible Electronics. Adv. Mater. Technol..

[182] Sun X., Yao F., Li J. (2020). Nanocomposite hydrogel-based strain and pressure sensors: A review. J. Mater. Chem. A.

[183] Lee Y.H., Wang L.Y., Tsai C.Y., Lee C.W. (2022). Self-healing nanocomposites with carbon nanotube/graphene/Fe3O4 nanoparticle tricontinuous networks for electromagnetic radiation shielding. ACS Appl. Nano Mater..

[184] Vu V.P., So H.M., Kim A., Lee J.Y., Oh M., Hyun S. (2025). Self-healing polymer binders: Next-generation battery applications. J. Mater. Chem. A.

[185] Gayathri K., Mahamani A., Basha J.S., Prakash A., Roshith P. (2025). Hybrid Nanocomposites for High-Performance Applications in Aerospace, Mechanical, and Biomedical Engineering Enhanced by Computational Modeling and AI. Advanced Materials for Biomedical Devices.

[186] Brochu A.B., Craig S.L., Reichert W.M. (2011). Self-healing biomaterials. J. Biomed. Mater. Res. Part A.

[187] Paolillo S., Bose R.K., Santana M.H., Grande A.M. (2021). Intrinsic self-healing epoxies in polymer matrix composites (PMCs) for aerospace applications. Polymers.

[188] Siwal S.S., Zhang Q., Devi N., Thakur V.K. (2020). Carbon-based polymer nanocomposite for high-performance energy storage applications. Polymers.

[189] Simões S. (2024). High-performance advanced composites in multifunctional material design: State of the art, challenges, and future directions. Materials.

[190] Babatunde D.E., Denwigwe I.H., Babatunde O.M., Agboola O., Akinsipe G.D. (2021). Relevance of chemically functionalized nano-fillers and modified nanocomposite in energy systems. Research Anthology on Synthesis, Characterization, and Applications of Nanomaterials.

[191] Kausar A. (2024). Shape Memory Polymer-Derived Nanocomposites: Materials, Properties, and Applications.

[192] Islam M.A., Talukder L., Al M.F., Sarker S.K., Muyeen S.M., Das P., Hasan M.M., Das S.K., Islam M.M., Islam M.R. (2023). A review on self-healing featured soft robotics. Front. Robot. AI.

[193] Chen Y., Zhao X., Li Y., Jin Z.Y., Yang Y., Yang M.B., Yin B. (2021). Light-and magnetic-responsive synergy controlled reconfiguration of polymer nanocomposites with shape memory assisted self-healing performance for soft robotics. J. Mater. Chem. C.

[194] Cobos C.G., Yan X., Martín-González M. (2025). 3D Polymeric Nanonetworks: From Self-Assembly to Advanced Fabrication. Macromol. Mater. Eng..

[195] Clayson I.G., Hewitt D., Hutereau M., Pope T., Slater B. (2020). High throughput methods in the synthesis, characterization, and optimization of porous materials. Adv. Mater..

[196] Wang P., Hu M., Wang H., Chen Z., Feng Y., Wang J., Ling W., Huang Y. (2020). The evolution of flexible electronics: From nature, beyond nature, and to nature. Adv. Sci..

[197] Liu X. (2025). Review of protective coatings for corrosion mitigation in chemical machinery: Performance and mechanical aspects. J. Adhes. Sci. Technol..

[198] Liu J., Urban M.W. (2024). Dynamic interfaces in self-healable polymers. Langmuir.

[199] Sahraei A.A., Mokarizadeh A.H., George D., Rodrigue D., Baniassadi M., Foroutan M. (2019). Insights into interphase thickness characterization for graphene/epoxy nanocomposites: A molecular dynamics simulation. Phys. Chem. Chem. Phys..

[200] Sarabia-Vallejos M.A., Cerda-Iglesias F.E., Pérez-Monje D.A., Acuña-Ruiz N.F., Terraza-Inostroza C.A., Rodríguez-Hernández J., González-Henríquez C.M. (2023). Smart polymer surfaces with complex wrinkled patterns: Reversible, non-planar, gradient, and hierarchical structures. Polymers.

[201] Sun H., Zhang T., Yin C., Sun H., Zhang C., Zhang Y., Zhang Y., Tang C., Chi Q. (2024). Significantly enhanced high-temperature energy storage performance for polymer composite films with gradient distribution of organic fillers. Chem. Eng. J..

[202] Patra T.K. (2021). Data-driven methods for accelerating polymer design. ACS Polym. Au.

[203] Sahu R.C., Arora S., Kumar D., Agrawal A.K. (2025). Machine Learning for Predictive Modeling in Nanomedicine-Based Cancer Drug Delivery. Med. Res..

[204] Madani S.S., Shabeer Y., Fowler M., Panchal S., Chaoui H., Mekhilef S., Dou S.X., See K. (2025). Artificial intelligence and digital twin technologies for intelligent lithium-ion battery management systems: A comprehensive review of state estimation, lifecycle optimization, and cloud-edge integration. Batteries.

[205] Rupanty N.S., Ghosh J., Noor T., Asif T.R., Ahmed S., Howlader S., Reukov V. (2025). Advances in Wearable Technology: MXene-Based Multifunctional and Biomedical Smart Textiles. ACS Omega.

[206] Hao Y., Zhu G. (2025). The Latest Advances in Mechanically Robust Self-Healing Polyurea Based on Dynamic Chemistry. Adv. Sci..

[207] Olonisakin K., Fan M., Xin-Xiang Z., Ran L., Lin W., Zhang W., Wenbin Y. (2022). Key improvements in interfacial adhesion and dispersion of fibers/fillers in polymer matrix composites; focus on pla matrix composites. Compos. Interfaces.

[208] Zeng Q., Liu H., Zhang Y., Zheng Y., Ding X., Zhu M., Shi G., Wang Y., Haick H., Zhang M. (2026). Materials and System Design for Self-Decision Bioelectronic Systems. Adv. Mater..

[209] Boahen E.K., Kong Z., Kim S.Y., Oh H., Yoo H., Lim J.S., Shin H.J., Kim J.H., Kim D.H. (2025). Autonomous Polymer Frameworks for Sustainable Tissue-Interfaced Plastic Bioelectronics. Adv. Sci..

[210] Saha R., Munshi M.H., Akter M., Shikder A.A.R., Islam T. (2025). Synthesis techniques, fundamental properties, and emerging applications of nanocomposites: A comprehensive review. SPE Polym..

[211] Duncan T.V. (2015). Release of engineered nanomaterials from polymer nanocomposites: The effect of matrix degradation. ACS Appl. Mater. Interfaces.

[212] Liu C., Fang W., Cheng Q., Qiu B., Shangguan Y., Shi J. (2025). Revolutionizing elastomer technology: Advances in reversible crosslinking, reprocessing, and self-healing applications. Polym. Rev..

[213] Paxton N.C., Zhao J., Sauret E. (2024). Polymer 3D printing in perspective: Assessing challenges and opportunities in industrial translation against the metal benchmark. Int. J. Adv. Manuf. Technol..

[214] Khan A., Ahmed N., Rabnawaz M. (2020). Covalent adaptable network and self-healing materials: Current trends and future prospects in sustainability. Polymers.

[215] Lucas S.S., Von Tapavicza M., Schmidt A.M., Bertling J., Nellesen A. (2016). Study of quantification methods in self-healing ceramics, polymers and concrete: A route towards standardization. J. Intell. Mater. Syst. Struct..

[216] Ouyang Q., Liu L., Wu Z. (2022). Electrothermally self-healing delamination cracks in carbon/epoxy composites using sandwich and tough carbon nanotube/copolymer interleaves. Polymers.

[217] Zhang T., Li W., Shi Y., Li C. (2022). Polyaniline-based room temperature ammonia gas sensor employing hybrid organic-inorganic substrate. Mater. Chem. Phys..

[218] He X., Cui C., Chen Y., Zhang L., Sheng X., Xie D. (2024). MXene and polymer collision: Sparking the future of high-performance multifunctional coatings. Adv. Funct. Mater..

[219] Joshi N.C., Gururani P., Gairola S.P. (2022). Metal oxide nanoparticles and their nanocomposite-based materials as photocatalysts in the degradation of dyes. Biointerface Res. Appl. Chem..

[220] Leporatti S. (2019). Polymer clay nano-composites. Polymers.

[221] Pati S., Singh B.P., Dhakate S.R. (2017). Self-healing polymer composites based on graphene and carbon nanotubes. Smart Polymer Nanocomposites: Energy Harvesting, Self-Healing and Shape Memory Applications.

